# A Circular RNA Promotes Tumor Metastasis through Stabilizing MSI2 Protein in Pancreatic Ductal Adenocarcinoma

**DOI:** 10.34133/research.0918

**Published:** 2025-10-03

**Authors:** Zhang Li, Lanyang Gao, Yang Yang, Yue Ming, Wenrong Liu, Tingting Zhang, Zixia Ye, Fuyan Xu, Juan He, Jiao Li, Jiawei Guo, Xiaojuan Yang, Qing Zhu, Yong Peng

**Affiliations:** ^1^ Center for Molecular Oncology, Frontiers Science Center for Disease-related Molecular Network, State Key Laboratory of Biotherapy and Cancer Center, State Key Laboratory of Respiratory Health and Multimorbidity, West China Hospital, Sichuan University, Chengdu 610041, China.; ^2^ Division of Abdominal Tumor Multimodality Treatment, Cancer Center, Department of General Surgery, West China Hospital, Sichuan University, Chengdu 610041, China.; ^3^ Frontiers Medical Center, Tianfu Jincheng Laboratory, Chengdu 610212, China.

## Abstract

Tumor metastasis is a major contributor for mortality in patients with pancreatic ductal adenocarcinoma (PDAC). While circular RNAs (circRNAs) have emerged as pivotal regulators of tumor progression and metastasis, their functional roles in PDAC remain poorly understood. Through comprehensive circRNA profiling of 10 pairs of PDAC tumors and adjacent normal tissues, we identified circPRKD3 (hsa_circ_0000992) as being substantially up-regulated in malignant specimens. Functional characterization demonstrated that circPRKD3 overexpression potently enhanced cellular migration, invasion, and metastatic capacity in vitro and in vivo, whereas its knockdown produced opposite phenotypic effects. Mechanistic investigations revealed that circPRKD3 directly interacted with the oncogenic RNA-binding protein Musashi-2, protecting it from β-TRCP-mediated ubiquitination and subsequent proteasomal degradation. Clinical correlation analysis revealed a close association between elevated circPRKD3 expression and shorter survival of PDAC patients. Notably, we validated the translational potential of circPRKD3 as a liquid biopsy marker, showing that serum detection, when combined with conventional biomarkers (CA19-9, CEA, and CA125), dramatically improved diagnostic performance. These findings not only delineate a novel circRNA-mediated regulatory axis in PDAC metastasis but also identify circPRKD3 as a promising diagnostic and prognostic biomarker.

## Introduction

Pancreatic ductal adenocarcinoma (PDAC) represents one of the most aggressive malignancies, with a 5-year survival rate below 10% [1]. While 15% to 20% of PDAC cases meet the surgical resection criteria at diagnosis, approximately 75% experience recurrence within 2 years post-resection, primarily attributable to undetected micro-metastatic lesions at the time of surgery [2]. A major factor underlying PDAC’s lethality is the difficulty of early detection, which is usually diagnosed after distant metastasis has already developed [3]. Importantly, emerging evidence demonstrates that PDAC cells disseminate into the circulation prior to forming detectable local invasions [4], highlighting the critical need for sensitive and reliable biomarkers capable of predicting metastatic potential at early disease stages.

Circular RNAs (circRNAs) are a unique class of endogenous RNA molecules characterized by covalently closed loop structures generated through back-splicing, wherein the 3′ splice donor of an upstream exon ligates to the 5′ splice acceptor of a downstream exon in precursor mRNAs [5]. These evolutionarily conserved molecules exhibit remarkable stability and abundance across eukaryotic cells, with accumulating evidence demonstrating their dysregulation in diverse pathological conditions [6,7]. In cancer biology, circRNAs have emerged as critical regulators of tumorigenesis and metastatic progression, while their inherent stability in biofluids renders them as promising candidates for liquid biopsy development [8,9]. Functional studies have established that circRNAs can exert potent oncogenic activities, as exemplified by circACC1-mediated enhancement of glycolytic metabolism to fuel tumor growth [10]*.* Conversely, our previous work revealed that the tumor-suppressive circNEIL3 inhibits tumor metastasis by recruiting the E3 ubiquitin ligase Nedd4L to facilitate degradation of the oncogenic protein YBX1 [11]. Although transcriptomic studies have identified numerous dysregulated circRNAs in PDAC [12], only a few have been functionally characterized [13]. This knowledge gap motivated us to investigate the roles of novel circRNAs in PDAC pathogenesis.

RNA-binding proteins (RBPs) function as master regulators of gene expression by controlling RNA metabolism, including splicing, stability, and translation. Imbalanced coordination between RBPs and their cognate RNA networks has been causally implicated in oncogenesis [14]. A paradigmatic example is Musashi-2 (MSI2), an oncogenic RBP that governs multiple hallmarks of cancer through modulation of pivotal signaling pathways [15]. MSI2’s transformative capacity is substantiated by its tumor-initiating potential in colorectal carcinogenesis, where its ectopic expression drives intestinal epithelial transformation in murine models [16]. Furthermore, elevated MSI2 expression drives oncogenesis and facilitates distant dissemination across multiple malignancies, including leukemias [17,18], non-small cell lung cancer (NSCLC) [19] and breast cancer [20]. In PDAC, MSI2 is substantially up-regulated and functionally validated to enhance tumor growth and metastasis in both ex vivo and in vivo models [21–23]. However, the precise molecular mechanisms driving MSI2 up-regulation remain elusive.

In this study, we identified circPRKD3 (hsa_circ_0000992) as a markedly up-regulated circRNAs in PDAC tumors. Functional characterization demonstrated that circPRKD3 overexpression potently enhanced PDAC cell migration, invasion, and metastatic capacity in both in vitro and in vivo models, whereas its knockdown exhibited the opposite phenotypic effects. Mechanistically, circPRKD3 directly interacted with MSI2, competitively disrupting the MSI2–β-TRCP interaction and thereby inhibiting ubiquitin-mediated degradation of MSI2. Clinically, elevated circPRKD3 expression in PDAC tumors correlated with poorer patient survival. Furthermore, we established the diagnostic potential of circPRKD3 in patient serum, where its detection synergized with conventional biomarkers (CA19-9, CEA, and CA125) to dramatically improve diagnostic accuracy. Collectively, these findings establish circPRKD3 as a critical regulator of PDAC metastasis through MSI2 stabilization and a promising noninvasive biomarker for PDAC diagnosis and prognosis.

## Results

### Up-regulation and characterization of circPRKD3 in PDAC

**Fig. 1.**
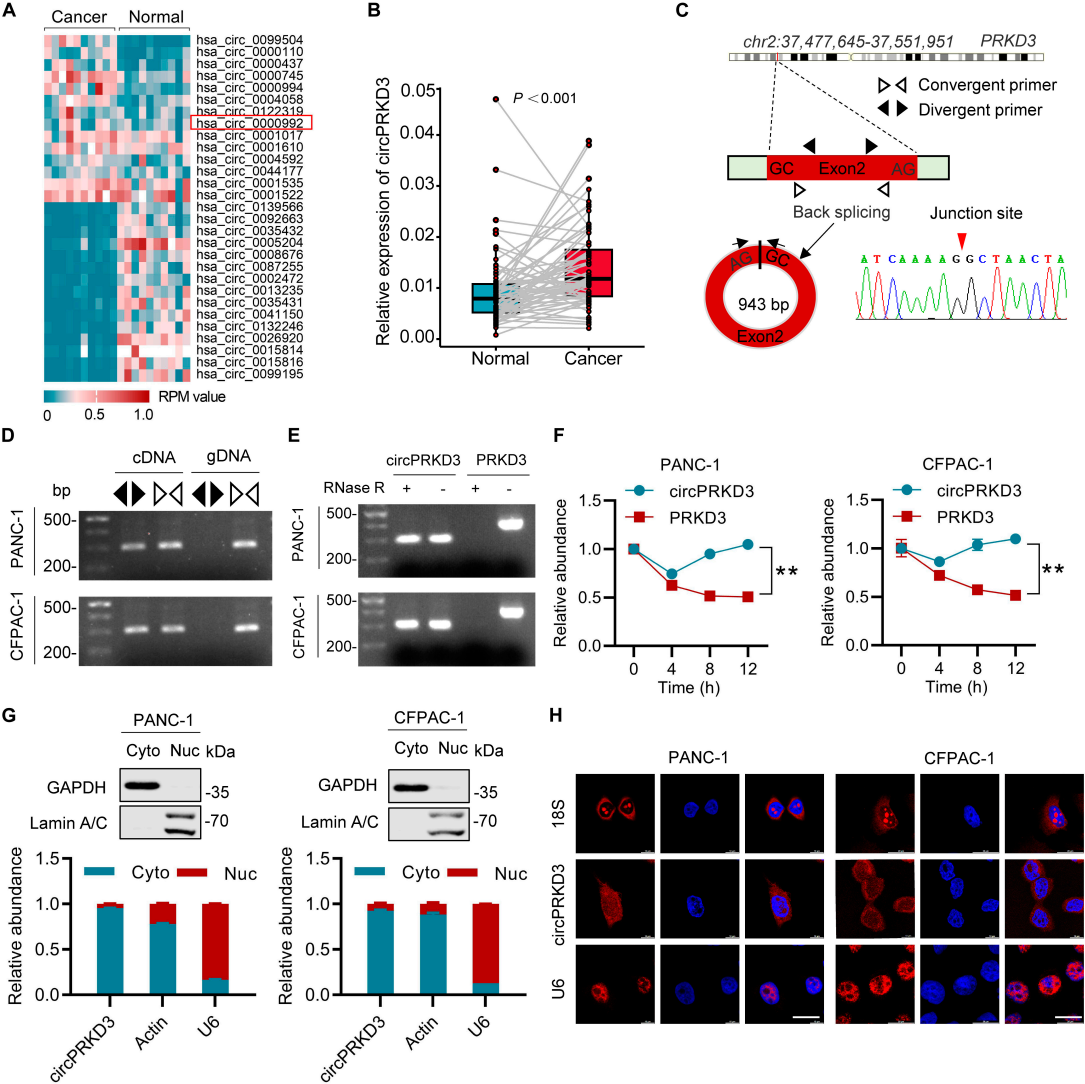
Up-regulation and characterization of circPRKD3 in PDAC. (A) Heatmap depicting differentially expressed circRNAs in 10 paired PDAC tumors and adjacent normal tissues. (B) RT-qPCR analysis demonstrating circPRKD3 up-regulation in PDAC tumors (*n* = 68). (C) Genomic locus of circPRKD3 with back-splicing junction validated by Sanger sequencing. (D) Detection of circPRKD3 and *PRKD3* mRNA using divergent (black triangle) and convergent (white triangle) primers in cDNA and gDNA templates. (E) RT-PCR analysis of circPRKD3 or *PRKD3* mRNA from total RNAs with or without RNase R treatment. (F) Time-course analysis of circPRKD3 and *PRKD3* mRNA stability following Actinomycin (2 μg/ml) treatment. (G) Subcellular distribution of circPRKD3 was evaluated by RT-qPCR, with *β-actin* mRNA and U6 snRNA as cytoplasmic (Cyto) and nuclear (Nuc) markers. Immunoblot analysis controls: GAPDH for cytoplasm and Lamin A/C for nucleus. (H) RNA FISH analysis of circPRKD3 in PANC-1 and CFPAC-1 cells. Nuclei were stained with DAPI (blue). 18S rRNA (cytoplasmic) and U6 snRNA (nuclear) served as localization controls. Scale bar = 20 μm. **P < 0.01.

To comprehensively identify dysregulated circRNAs in PDAC, we conducted Ribo-Zero RNA sequencing of 10 matched tumor-normal tissue pairs. This systematic screening identified 71 circRNAs with marked expression alterations (Table S1), with hierarchical clustering revealing the top 14 up-regulated and 15 down-regulated candidates (Fig. 1A). Through functional screening, we prioritized circPRKD3 (hsa_circ_0000992) as a top metastasis-associated candidate, ranking among the 10 most up-regulated circRNAs (Fig. S1A). Independent validation by reverse transcription quantitative polymerase chain reaction (RT-qPCR) analysis in an expanded cohort (*n* = 68 matched pairs) confirmed its significant up-regulation (Fig. 1B). Molecular characterization demonstrated that this 943-nucleotide circRNA arises from back-splicing of exon 2 in the *PRKD3* gene (Fig. 1C). This biogenesis mechanism was further confirmed by the observation that divergent primer amplification specifically detected circPRKD3 in complementary DNA (cDNA) templates, but not in genomic DNA (gDNA) of PANC-1 and CFPAC-1 cells, while convergent primers successfully amplified products from both cDNA and gDNA templates (Fig. 1D). Biochemical analyses showed that circPRKD3 exhibits resistance to RNase R digestion (Fig. 1E) and enhanced stability compared to linear *PRKD3* mRNA (Fig. 1F). Subcellular fractionation (Fig. 1G) and RNA fluorescence in situ hybridization (FISH) analyses (Fig. 1H) consistently revealed circPRKD3’s predominant cytoplasmic localization in PDAC cells. Together, these results indicate that circPRKD3 is a bona fide circRNA that is substantially overexpressed in PDAC.

### Enforced circPRKD3 expression promotes tumor metastasis in PDAC

**Fig. 2.**
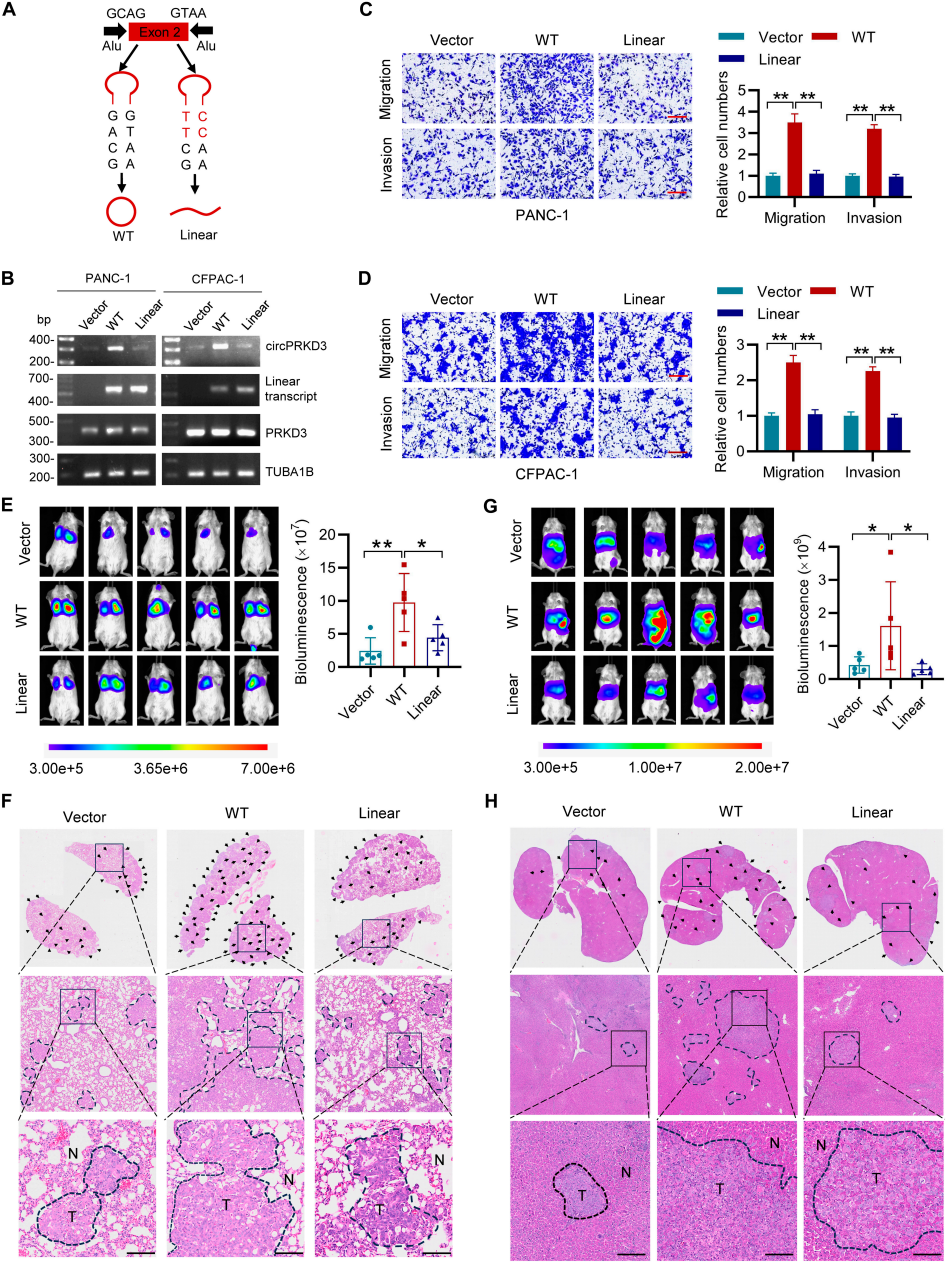
Enforced circPRKD3 expression promotes PDAC metastasis. (A) Design of wild-type (WT) circPRKD3 expression vector and its linear transcript mutant (Linear). (B) Agarose gel electrophoresis of RT-PCR products demonstrating successful circularization of WT circPRKD3 but not linear mutant transcripts. (C and D) Transwell assays showing enhanced migratory and invasive capacities of PANC-1 (C) and CFPAC-1 (D) cells overexpressing WT circPRKD3 (but not Linear mutant) compared to empty vector controls. Scale bar = 250 μm. (E and F) Lung metastatic model: NSIG mice (*n* = 5 per group) intravenously injected with 1 × 10^6^ luciferase-labeled CFPAC-1 cells expressing vector (Vector), WT circPRKD3, or Linear mutant. In vivo bioluminescence imaging and quantification of lung metastasis are shown in (E), and the representative H&E-stained lung sections show metastatic nodules (F). T: tumor; N: normal; arrows indicate lesions. Scale bar = 100 μm. (G and H) Liver metastatic model: NSIG mice (*n* = 5 per group) receiving intrasplenic injection of 1 × 10^6^ luciferase-labeled PANC-1 cells. In vivo bioluminescence imaging and quantification of liver metastasis are shown in (G), and the representative H&E-stained liver sections showed metastatic foci (H). Scale bar = 100 μm. *P < 0.05, **P < 0.01.

To elucidate the functional role of circPRKD3 in PDAC pathogenesis, we first characterized its endogenous expression patterns across multiple PDAC cell lines (Fig. S1B) and selected CFPAC-1 and PANC-1 cells for gain-of-function studies. Successful circularization and cytoplasmic localization of exogenous cricRPDK3 were validated by Sanger sequencing and RT-qPCR analyses (Fig. S1C to E). We further generated a structural mutant by introducing AG→TT and GT→CC substitutions in the circularization elements of the wild-type (WT) cricPRKD3 expression plasmid (Fig. 2A), which exclusively expressed the linear transcripts but not circPRKD3 (hereafter designated “Linear” mutant; Fig. 2B). Functional characterization showed that while WT circPRKD3 overexpression minimally impacted cellular proliferation and clonogenic potential (Fig. S1F and G), it dramatically enhanced migratory and invasive capacities in both cell models (Fig. 2C and D and Fig. S1H and I). Importantly, the Linear mutant failed to recapitulate these pro-metastatic effects, demonstrating the structure-dependent functional specificity of circPRKD3 in promoting PDAC migration and invasion.

To investigate the in vivo metastatic potential of circPRKD3, we employed 2 distinct xenograft models in NOD-Prkdc^scid^ IL2rg^tm1^ (NSIG) mice. In the lung metastasis model, intravenous injection of CFPAC-1 cells revealed that WT circPRKD3 substantially increased pulmonary metastatic lesions compared to the vector controls, while the Linear mutant showed no significant effect, as quantified by bioluminescent imaging (Fig. 2E) and histologically confirmed by hematoxylin and eosin (H&E) staining (Fig. 2F). Similarly, in the liver metastasis model by injection of PANC-1 cells into the spleen, WT cirPRKD3 markedly enhanced hepatic metastasis formation, whereas the Linear mutant failed to promote metastatic dissemination (Fig. 2G and H). To specifically identify human PDAC metastases within murine tissues, we performed immunohistochemistry (IHC) staining for nucleolin, a well-characterized marker of PDAC proliferation and survival [24,25]. Quantitative analysis demonstrated that WT circPRKD3-expressing tumors generated more nucleolin-positive metastatic foci compared to both vector controls and Linear mutant groups (Fig. S2A and B). Collectively, these in vivo findings indicate that WT circPRKD3, but not its linear transcript, promotes PDAC metastasis.

To explore the molecular mechanisms underlying circPRKD3-mediated PDAC progression, we performed transcriptome sequencing to identify differentially expressed genes in circPRKD3-overexpressing PANC-1 cells. Bioinformatics analysis identified the top 10 enriched Gene Ontology (GO) pathways with the smallest *P* values, with focal adhesion and cell–substrate junction pathways demonstrating the strongest association (Fig. S2C). Gene Set Enrichment Analysis (GSEA) further revealed that circPRKD3 overexpression was associated with activation of established pro-metastatic pathways, including focal adhesion, epithelial–mesenchymal transition (EMT), MYC targets, and MAPK (mitogen-activated protein kinase) signaling pathway (Fig. S2D). Notably, circPRKD3 did not alter protein levels of EMT markers (E-cadherin, N-cadherin, and Vimentin; Fig. S2E). We therefore focused on focal adhesion-related genes, which showed significant enrichment. RT-qPCR analysis confirmed consistent up-regulation of focal adhesion components (*FLNA*, *ITGB1*, *LAMB3*, and *PXN* mRNAs) in circPRKD3-overexpressing PDAC cells (Fig. S2F). These results suggest that circPRKD3 promotes metastasis through focal adhesion-mediated mechanisms rather than through classical EMT regulation.

### CircPRKD3 depletion suppresses tumor metastasis in PDAC

**Fig. 3.**
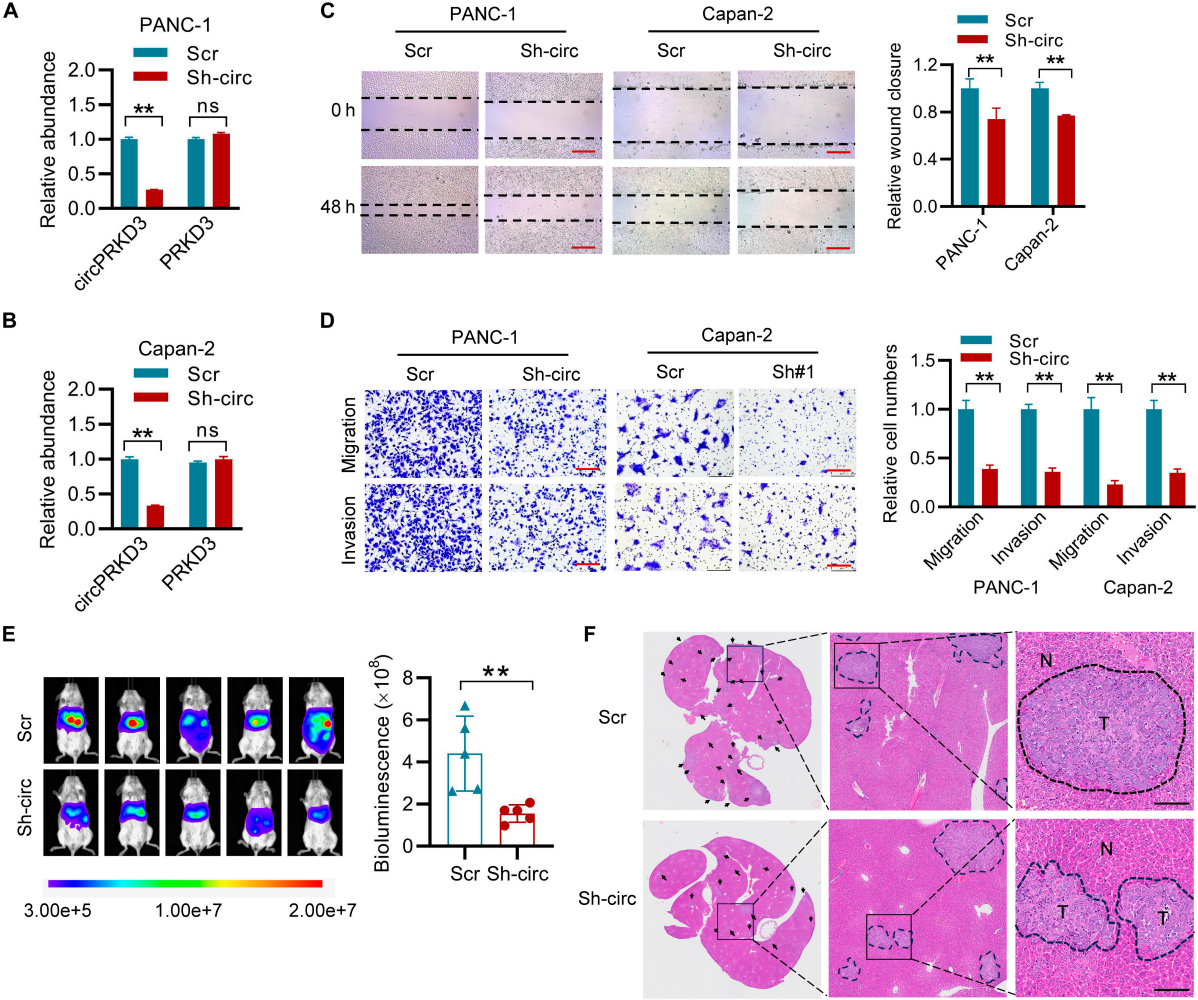
Genetic depletion of circPRKD3 suppresses PDAC metastasis. (A and B) Validation of circPRKD3 knockdown efficiency by RT-qPCR in PANC-1 (A) and Capan-2 (B) cells transduced with shRNA targeting circPRKD3 (Sh-circ) versus nontargeting scramble control (Scr). (C) Wound-healing assays showing impaired cell motility in Sh-circ transduced cells compared to controls at 48 h post-wounding. Scale bar = 200 μm. (D) Transwell migration and Matrigel invasion assays revealing reduced metastatic potential in circPRKD3-depleted cells. Scale bar = 250 μm. (E and F) Liver metastasis model: NSIG mice (*n* = 5 per group) receiving intrasplenic injection of 1 × 10^6^ luciferase-labeled PANC-1 cells with Sh-circ or Scr. In vivo bioluminescence imaging and quantification of hepatic metastases at endpoint are shown in (E), and the representative H&E-stained liver sections showing metastatic burden (F). Scale bar = 100 μm. **P < 0.01.

The functional significance of circPRKD3 in PDAC was further validated by the loss-of-function strategy using short-hairpin RNA (shRNA) targeting the junction sequence of circPRKD3 (Sh-circ). RT-qPCR analysis confirmed the specific and efficient knockdown of circPRKD3 in both PANC-1 and Capan-2 cell lines, without affecting *PRKD3* mRNA expression (Fig. 3A and B and Fig. S3A). Functional assays demonstrated that circPRKD3 depletion substantially impaired wound healing capacity, Transwell migration, and Matrigel invasion in PDAC cells (Fig. 3C and D), while exhibiting no apparent effect on cellular proliferation or clonogenic potential (Fig. S3B and C). Notably, although immunoblot analysis detected no alternations in classical EMT markers (Fig. S3D), circPRKD3 silencing dramatically down-regulated focal adhesion-related transcripts (*FLNA*, *ITGB1*, *LAMB3*, and *PXN*; Fig. S3E). Consistent with these in vitro findings, in vivo evaluation using a liver metastasis model demonstrated that circPRKD3 knockdown markedly decreased hepatic metastatic burden compared to scramble controls (Scr), as quantified by bioluminescent imaging (Fig. 3E) and confirmed through histopathological analyses (H&E staining and anti-nucleolin IHC; Fig. 3F and Fig. S3F). Together, these findings demonstrate that circPRKD3 depletion effectively suppresses tumor metastasis in PDAC.

### CircPRKD3 physically interacts with MSI2 in PDAC cells

Current evidence demonstrates that circRNAs mediate their biological functions through multiple molecular mechanisms, including microRNA sponge, protein sequestration, or peptide translation [26,27]. Our bioinformatics analyses using TransCirc [28] and riboCIRC [29] predicted that circPRKD3 has limited protein-coding potential and minimal microRNA sponge capacity. This finding led us to hypothesize that circPRKD3 likely mediates its oncogenic functions through protein interactions.

**Fig. 4.**
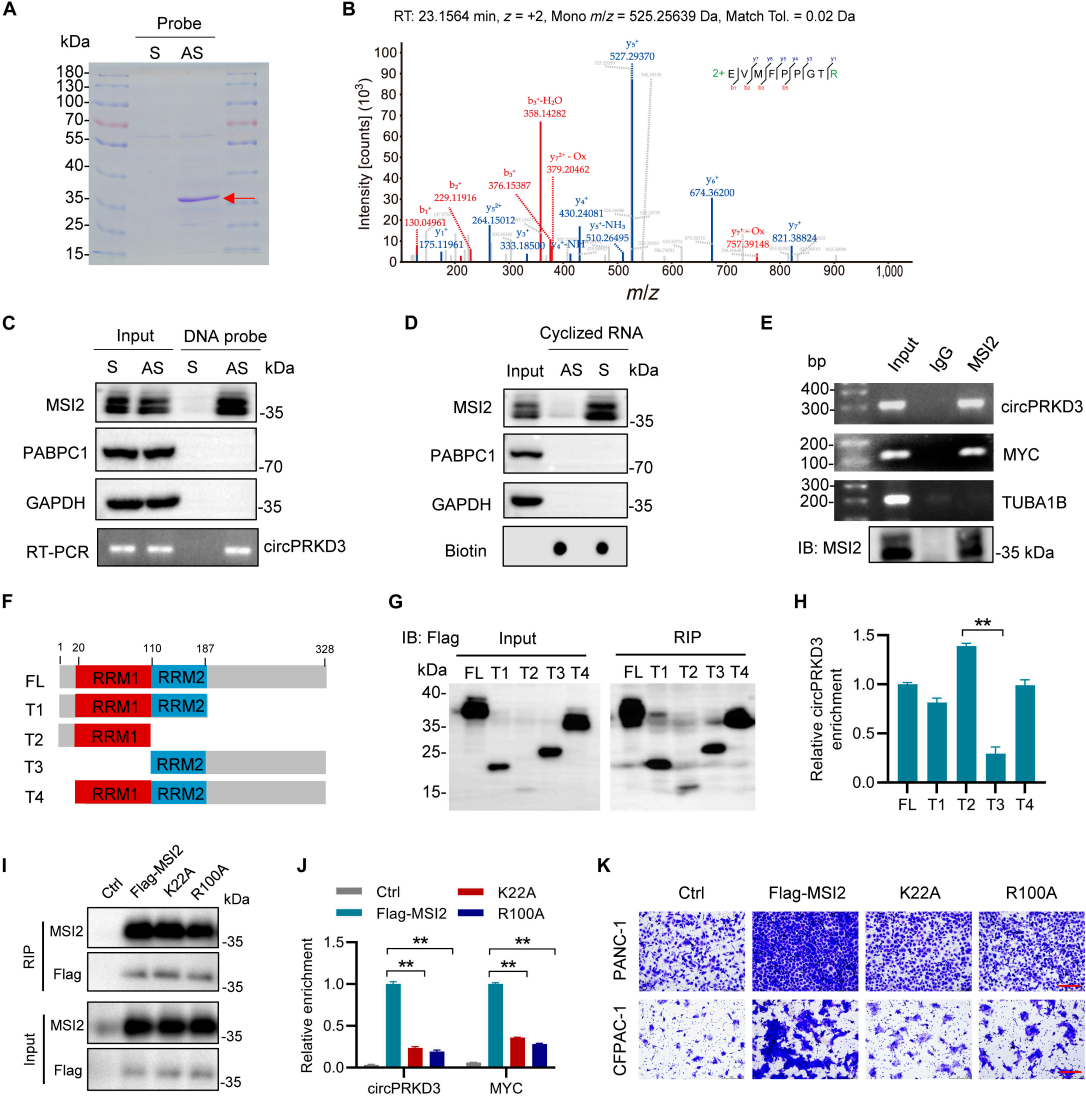
CircPRKD3 physically interacts with MSI2 in PDAC cells. (A) Coomassie blue staining of cytoplasmic proteins pulled down by circPRKD3 antisense (AS) or sense (S) DNA probes in PANC-1 cells. (B) Representative MS/MS spectrum identifying the MSI2 peptide from MS analysis of the excised band. (C) RNA pull-down assays showing specific association of MSI2 with circPRKD3, as confirmed by immunoblot analysis (top) and RT-PCR detection of circRNA (bottom). PABPC1 and GAPDH proteins serve as nonspecific controls. (D) In vitro circularized sense strand (S) circPRKD3 specifically enriches endogenous MSI2 proteins from PANC-1 cell lysates. Probe loading was verified by streptavidin-HRP. (E) RIP assays demonstrating enrichment of circPRKD3 by anti-MSI2 antibody compared to IgG control. *MYC* mRNA and *TUBA1B* mRNA serve as positive and negative controls, respectively. (F) Schematic diagrams of MSI2 RNA-binding domains and truncated mutants. (G and H) Flag-RIP experiments were performed in PANC-1 cells expressing MSI2 truncated mutants. Expression and IP efficiency of MSI2 mutants were evaluated by immunoblot analysis using anti-Flag antibody (G). CircPRKD3 enrichment by MSI2 truncated mutants was measured by RT-qPCR analysis (H). (I and J) Flag-RIP assays conducted in PANC-1 cells expressing either WT MSI2 or RRM1 mutants. Immunoblot analysis with anti-Flag antibody confirmed expression and IP efficiency of MSI2 mutants (I). RT-qPCR quantification revealed reduced circPRKD3 enrichment by K22A or R100A mutants compared to WT MSI2 (J). *MYC* mRNA serves as positive controls. (K) Transwell migration assays demonstrated that K22A and R100A mutants impaired the pro-migratory effects of MSI2 in both PANC-1 and CFPAC-1 cells. **P < 0.01.

To identify proteins interacting with circPRKD3, we conducted RNA pull-down assays in PANC-1 cells using biotinylated DNA probes (sense and antisense, S and AS) specifically targeting the circPRKD3 back-splice junction. Based on circPRKD3’s predominant cytoplasmic localization in PDAC cells, cytoplasmic lysates were utilized for the interaction studies. Coomassie blue staining detected a ~37-kDa protein band specifically enriched by the antisense probe (Fig. 4A). Subsequent mass spectrometry (MS) analysis identified this band as MSI2, which ranked the top scoring RBPs among all captured proteins (Fig. 4B and Table S2).

The circPRKD3–MSI2 interaction was firstly validated by immunoblot analysis following RNA pull-down, which confirmed MSI2 enrichment by the antisense probe compared to the sense probe, with the RBP PABPC-1 serving as a negative control that showed no enrichment (Fig. 4C). Moreover, MSI2 was specifically pulled down by in vitro transcribed and circularized circPRKD3 (sense strand), but not by its antisense control (Fig. 4D). Reciprocal RIP (RNA-binding protein immunoprecipitation) assays showed significant enrichment of circPRKD3 by anti-MSI2 antibody compared to the IgG control (Fig. 4E), with *MYC* mRNA (a known MSI2 target [30,31]) and *TUBA1B* mRNA serving as positive and negative controls, respectively. To map the interaction domain, we generated Flag-tagged MSI2 truncation mutants containing either RRM1 and/or RRM2 domain (Fig. 4F). Flag-RIP assays demonstrated preferential binding of circPRKD3 to the RRM1 domain (Fig. 4G and H), establishing the structural basis for this RNA–protein interaction. Emerging evidence identifies K22 and R100 as critical residues for RNA binding in the MSI2 RRM1 domain [32]. To validate their functional importance, we generated K22A and R100A mutants and assessed their interaction with circPRKD3. Flag-RIP assays demonstrated reduced binding of both mutants to circPRKD3 and *MYC* mRNA (Fig. 4I and J). Consistent with this impaired RNA-binding capacity, these mutations attenuated MSI2’s migration-promoting effects in PDAC cells (Fig. 4K). Together, these biochemical analyses demonstrate the specific association of circRPKD3 with MSI2 in PDAC cells.

### CircPRKD3 promotes PDAC metastasis through its interaction with MSI2

MSI2 has emerged as a pivotal regulator of pancreatic cancer progression [21–23]. Our analysis of the GSE71729 dataset revealed substantially higher MSI2 expression in metastatic PDAC specimens versus primary tumors (Fig. S4A), with elevated MSI2 levels correlating with poorer survival of PDAC patients across multiple independent cohorts (GSE21501, GSE71729, and ICGC_PAAD_AU_array; Fig. S4B). To determine MSI2’s role in circPRKD3-mediated metastasis, we employed shRNA-mediated knockdown in circPRKD3-overexpressing PANC-1 and CFPAC-1 cells, achieving efficient MSI2 depletion (Fig. S4C). Notably, MSI2 silencing dramatically attenuated the promoting effects of circPRKD3 on PDAC cell migration and invasion (Fig. S4D and E), indicating that MSI2 is a key downstream effector of circPRKD3’s metastatic functions. MSI2 is known to regulate key epithelial motility factors, particularly focal adhesion components such as FLNA, ITGB1, and LAMB3 [33]. Consistent with this, MSI2 knockdown abolished circPRKD3’s up-regulation of focal adhesion-related transcripts (*FLNA*, *ITGB1*, *LAMB3*, and *PXN* mRNAs; Fig. S4F and G), suggesting that circPRKD3 promotes metastasis through MSI2-mediated modulation of focal adhesion pathways.

**Fig. 5.**
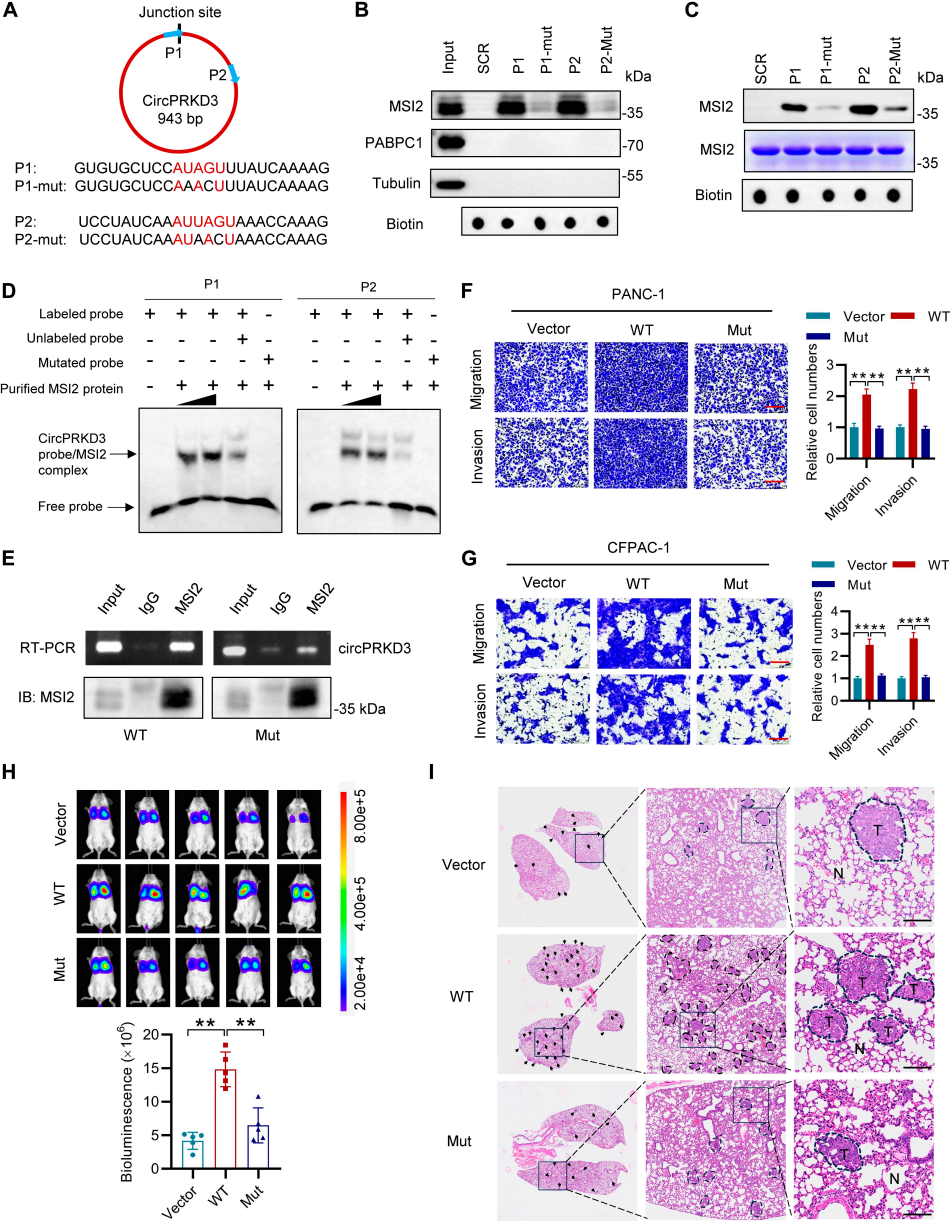
CircPRKD3 promotes PDAC metastasis through its interaction with MSI2 (A) Schematic diagram of 2 putative MSI2-binding regions (P1 and P2) containing the “UAGU” motifs (top) and sequences of WT (P1 and P2) and mutant (P1-mut and P2-mut) RNA probes (bottom). (B) RNA pull-down assays demonstrating MSI2 specifically binds to “UAGU”-containing P1/P2 probes in PANC-1 lysates. Both PABPC1 and tubulin serve as negative controls. Probe loading was verified by streptavidin-HRP. (C) RNA pull-down assays showing direct interaction between recombinant MSI2 proteins and biotinylated P1/P2 probes (but not mutants). (D) RNA-EMSAs confirming the direct binding of recombinant MSI2 protein with biotinylated P1/P2 probes. (E) RIP-qPCR showing MSI2 preferentially interacted with WT circPRKD3 (WT) over MSI2-binding-deficient mutants (Mut) in PDAC cells. (F and G) Transwell assays revealing that WT circPRKD3 but not MSI2-binding-deficient mutant (Mut) promoted migration and invasion of PANC-1 (F) and CFPAC-1 cells (G). Scale bar = 250 μm. (H and I) Lung metastasis model: NSIG mice (*n* = 5 per group) intravenously injected with 1 × 10^6^ luciferase-labeled CFPAC-1 cells expressing vector (Vector), WT circPRKD3, or MSI2-binding-deficient mutant (Mut). In vivo bioluminescence imaging and quantification of lung metastasis are shown in (H), and the representative H&E-stained lung sections showed metastatic nodules (I). Scale bar = 100 μm. T: tumor tissue; N: normal tissue. Arrows indicate metastatic lesions. **P < 0.01.

Previous studies established that MSI2 recognizes a conserved “UAGU” motif in target RNAs [22,33,34]. To define the molecular basis of the circPRKD3–MSI2 interaction, we identified 2 putative MSI2-binding regions in circPRKD3, each containing the “UAGU” sequence, and designed corresponding biotinylated RNA probes (P1 and P2; Fig. 5A). These regions harbor high-affinity binding sites (“AUUAGU” and “AUAGU”) that are shared with known MSI2 targets such as *BRD4*, *HMGA2*, and *cMET* mRNAs [22]. RNA pull-down assays showed that both WT probes (P1 and P2) efficiently precipitated MSI2 proteins from PDAC cell lysates (Fig. 5B). Mutation of the core “UAGU” motif to “AACU” (P1-mut and P2-mut) abolished MSI2 binding, indicating the essential role of this motif (Fig. 5B). Furthermore, RNA pull-down (Fig. 5C) and RNA-electrophoretic mobility shift assays (EMSAs) (Fig. 5D) using recombinant MSI2 protein verified the direct and sequence-specific nature of this interaction. Genetic validation using binding-site mutants in the circPRKD3 expression construct showed that while WT circPRKD3 was enriched by anti-MSI2 antibody, the MSI2-binding-deficient mutant (Mut) dramatically attenuated this interaction (Fig. 5E), confirming that circPRKD3 associates with MSI2 through the “UAGU” motifs.

To investigate whether cricPRKD3’s pro-metastatic effects depend on MSI2 binding, we performed functional characterization of PANC-1 and CFPAC-1 cells expressing either WT or Mut circPRKD3. While WT circPRKD3 robustly enhanced cellular migration and invasion, the circPRKD3-Mut failed to promote these metastatic properties (Fig. 5F and G). Consistently, circPRKD3-Mut showed impaired ability to up-regulate focal adhesion components (*FLNA*, *ITGB1*, *LAMB3*, and *PXN* mRNAs; Fig. S4H). Moreover, in vivo studies using a lung metastasis model demonstrated that CFPAC-1 cells expressing circPRKD3-Mut had metastatic potential comparable to vector controls, whereas WT-expressing cells showed substantially increased metastatic burden, as quantified by bioluminescent imaging (Fig. 5H) and confirmed through histopathology (H&E and anti-nucleolin staining; Fig. 5I and Fig. S4I). These findings definitely establish that circPRKD3 promotes PDAC metastasis through its specific interaction with MSI2.

### CircPRKD3 stabilizes MSI2 by inhibiting its ubiquitin-proteasome degradation

**Fig. 6.**
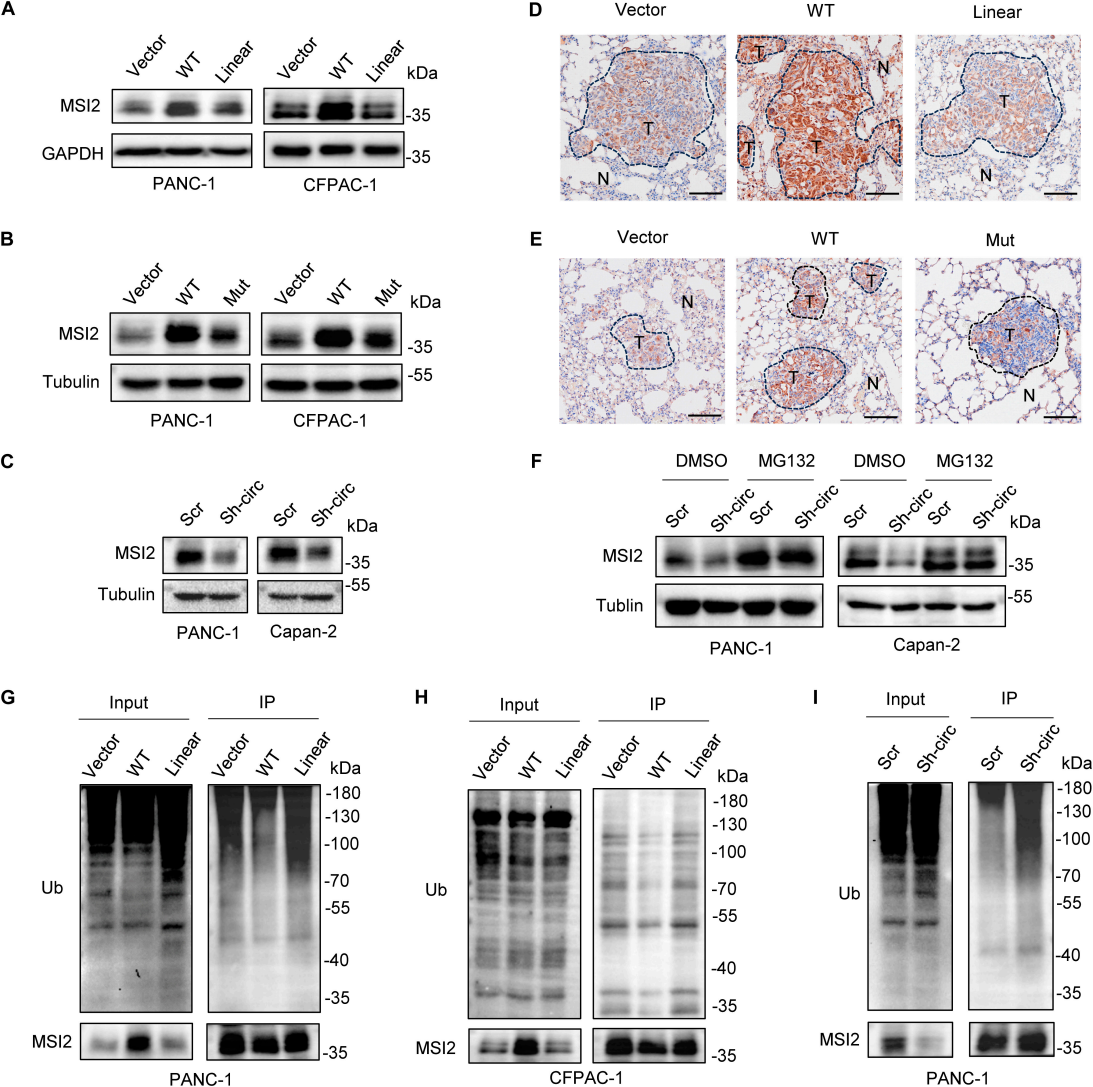
CircPRKD3 stabilizes MSI2 by inhibiting its ubiquitin-proteasome degradation. (A and B) Immunoblot analysis showing that WT circPRKD3, but not its linear transcript mutant (Linear) or MSI2-binding-deficient mutant (Mut), up-regulated MSI2 protein levels in PANC-1 and CFPAC-1 cells. (C) CircPRKD3 depletion reduced endogenous MSI2 protein expression in PANC-1 and Capan-2 cells. (D and E) IHC staining of MSI2 protein in lung metastatic lesions from mice injected with CFPAC-1 cells expressing with vector, WT circPRKD3, linear transcript mutant (D), or MSI2-binding site mutant (Mut) (E). Scale bar = 100 μm. T: tumor; N: normal tissue. (F) Proteasome inhibition with MG132 (20 μM, 6 h) restored MSI2 protein levels in circPRK3-depleted cells. (G and H) Ubiquitination assays showing reduced MSI2 polyubiquitination levels in cells overexpressing WT circPRKD3 compared to mutants. (I) Enhanced MSI2 polyubiquitination following circPRKD3 knockdown.

Emerging evidence indicates that circRNA–protein interactions can critically regulate protein stability and function [26]. Given our demonstration of direct circPRKD3–MSI2 binding, we investigated whether circPRKD3 modulates MSI2 homeostasis. Immunoblot analysis showed that WT circPRKD3 overexpression apparently increased MSI2 protein levels in both PANC-1 and CFPAC- cells, while neither the Linear transcript mutant (Linear) nor MSI2-binding-deficinet mutant (Mut) had this effect (Fig. 6A and B). Conversely, circPRKD3 depletion dramatically reduced MSI2 protein abundance (Fig. 6C). This regulation was further confirmed in vivo, where IHC analysis of lung metastases from NSIG mice showed stronger MSI2 staining in tumors from circPRKD3-WT-overexpressing cells compared to vector controls or circPRKD3-Mut-overexpressing cells (Fig. 6D and E). Notably, RT-qPCR analysis showed that neither circPRKD3 overexpression nor depletion affected *MSI2* mRNA levels (Fig. S5A and B). These findings collectively demonstrate that circPRKD3 regulates MSI2 protein stability through direct interaction.

To elucidate the mechanism underlying circPRKD3-mediated MSI2 stabilization, we employed the protease inhibitor MG132 to treat PDAC cells. The results showed that treatment with MG132 reversed the decrease in MSI2 protein levels caused by circPRKD3 knockdown (Fig. 6F), indicating that circPRKD3 protects MSI2 from proteasomal degradation. Subsequent ubiquitination assays revealed that overexpression of WT circPRKD3, but not the Linear transcript mutant, substantially decreased MSI2 polyubiquitination (Fig. 6G and H), while circPRKD3 depletion increased MSI2 polyubiquitination (Fig. 6I). Together, these findings demonstrate that circPRKD3 physically interacts with MSI2 to prevent its ubiquitin-proteasome-dependent degradation, thereby sustaining MSI2 protein stability in PDAC cells.

### CircPRKD3 disrupts the interaction between MSI2 and β-TRCP

**Fig. 7.**
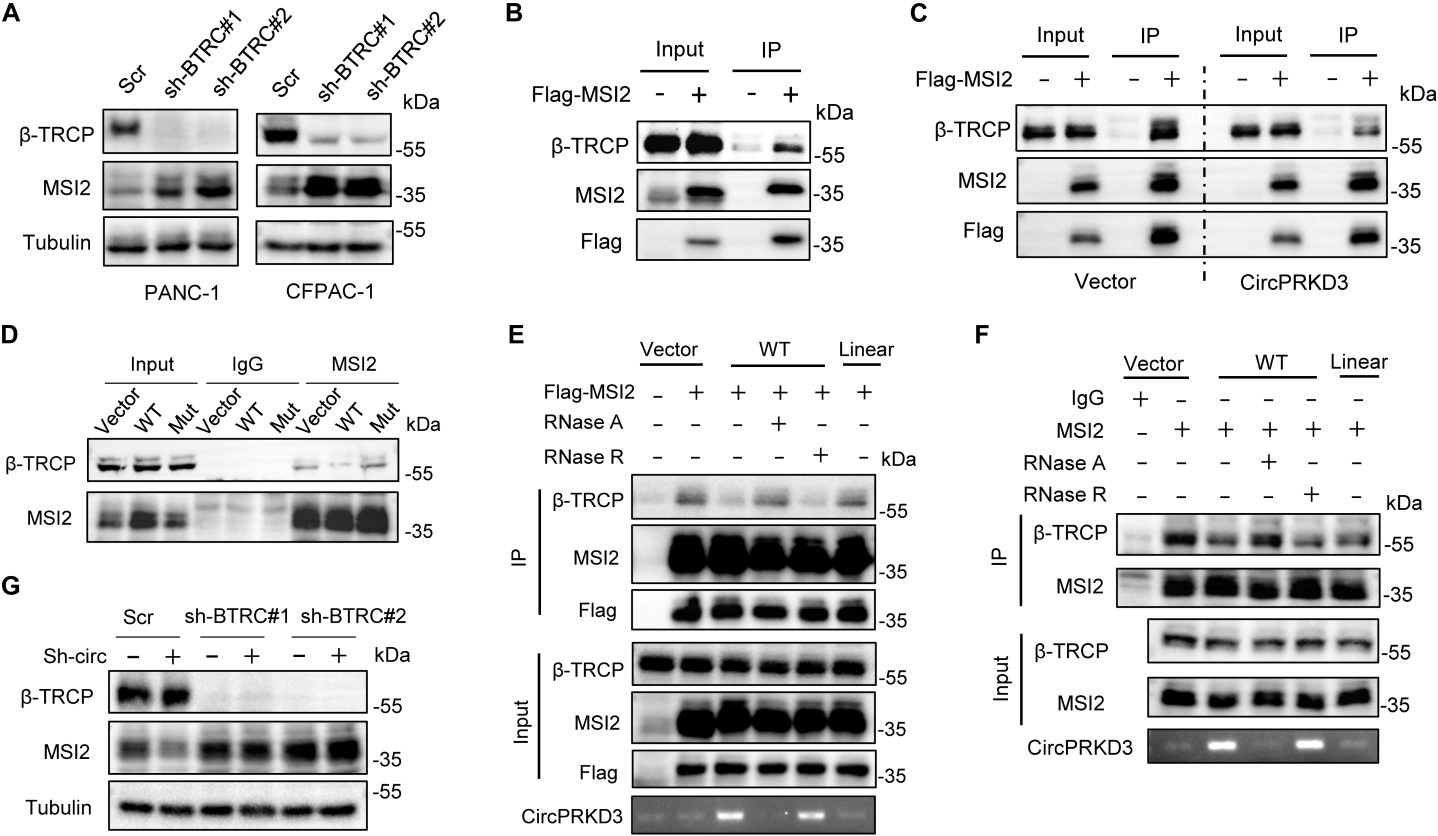
CircPRKD3 disrupts the interaction between MSI2 and β-TRCP. (A) Effects of β-TRCP knockdown on MSI2 expression in PANC-1 and CFPAC-1 cells. (B) Co-IP assays with anti-Flag antibody confirming physical interaction between Flag-MSI2 and β-TRCP in PANC-1 cells. (C) circPRKD3 overexpression reduced the MSI2–β-TRCP interaction in co-IP assays with anti-Flag antibody. (D) Co-IP assays with anti-MSI2 antibody showing that WT circPRKD3, but not MSI2-binding-deficient mutant (Mut), disrupted MSI2–β-TRCP complex formation in PANC-1 cells. (E and F) Co-IP assays showed that WT circPRKD3 decreased the association of β-TRCP with Flag-MSI2 (E) and endogenous MSI2 (F) compared with vector controls or linear transcript mutants (Linear). This inhibitory effect was abolished by RNase A treatment but remaining intact following RNase R digestion. (G) Depletion of circPRKD3 reduced MSI2 protein levels in PANC-1 cells, an effect that was rescued by concomitant β-TRCP knockdown.

To identify the E3 ubiquitin ligase responsible for MSI2 degradation in PDAC, we combined literature mining with our MS data from RNA pull-down assays. This approach identified 3 candidates: HUWE1 (from our MS data), and β-TRCP and DBC2 (previously reported to degrade MSI2 in gastric and breast cancer, respectively [30,35]). Functional screening using shRNA knockdown revealed that only β-TRCP depletion substantially increased MSI2 protein levels (Fig. 7A and Fig S6A). Moreover, co-immunoprecipitation (co-IP) assays confirmed a physical interaction between β-TRCP and MSI2 in PANC-1 cells (Fig. 7B). These results indicate that β-TRCP is the specific E3 ligase governing MSI2 stability in PDAC and suggest its involvement in circPRKD3-mediated stabilization of MSI2.

To determine how circPRKD3 regulates MSI2 stability, we first examined the potential interaction between circPRKD3 and β-TRCP. RNA pull-down assays using in vitro transcribed and circularized circPRKD3 demonstrated MSI2 enrichment but not β-TRCP (Fig. S6B), excluding a direct circPRKD3-β-TRCP binding. Since circPRKD3 overexpression reduces MSI2 ubiquitination, we hypothesized that circPRKD3 competitively inhibits MSI2–β-TRCP binding. To test this, we performed co-IP assays and found that WT circPRKD3 overexpression substantially reduced MSI2–β-TRCP complex formation in PDAC cells compared to vector control (Fig. 7C). Importantly, this effect was abolished when using MSI2-binding-deficient circPRKD3 mutant (Mut), which failed to disrupt MSI2–β-TRCP interaction (Fig. 7D). These results demonstrate that circPRKD3 physically competes with β-TRCP for MSI2 binding.

To further validate circPRKD3’s role in modulating the MSI2–β-TRCP interaction, we treated circPRKD3-overexpressing PDAC cells with RNase A (an RNA endonuclease) or RNase R (an RNA exonuclease) prior to co-IP assays. The results showed that treatment with RNase A (but not RNase R) dramatically attenuated circPRKD3-mediated disruption of MSI2–β-TRCP binding (Fig. 7E and F), consistent with circPRKD3’s known resistance to exonucleolytic cleavage. Importantly, the circPRKD3-dependent stabilization of MSI2 relied on β-TRCP, as β-TRCP knockdown abrogated MSI2 up-regulation (Fig. 7G). Furthermore, while circPRKD3 depletion suppressed PDAC cell migration and invasion, concomitant β-TRCP knockdown restored these metastatic properties to control levels (Fig. S6C). Taken together, these results demonstrate that circPRKD3 stabilizes MSI2 by physically competing with β-TRCP for MSI2 binding.

### Clinical significance of circPRKD3–MSI2 axis in PDAC

**Fig. 8.**
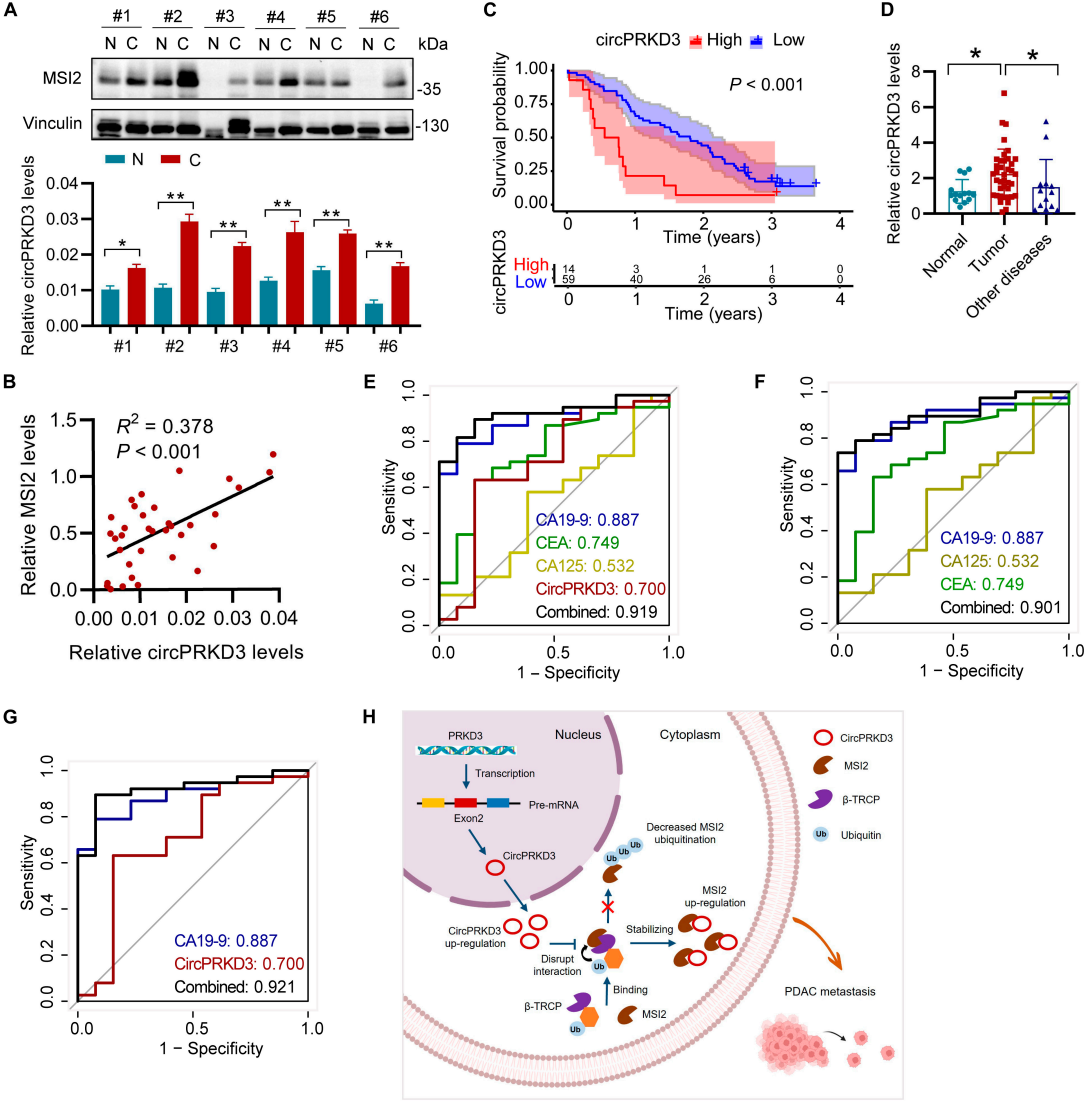
Clinical significance of the circPRKD3–MSI2 axis in PDAC. (A) MSI2 and circPRKD3 expression were measured by immunoblot (top) and RT-qPCR analysis (bottom) in paired PDAC tumors (C) and adjacent normal tissues (N). (B) Pearson correlation analysis between MSI2 protein and circPRKD3 levels in PDAC specimens. (C) Kaplan–Meier overall survival analysis of PDAC patients stratified by circPRKD3 expression. (D) RT-qPCR analysis of serum circPRKD3 levels in PDAC patients (Tumor) versus healthy controls (Normal) and patients with other pancreas-related diseases (Other diseases). (E and F) ROC analysis comparing the diagnostic performance of circPRKD3, conventional markers (CA19-9, CEA, and CA125), and combined panels for distinguishing PDAC from other pancreas-related diseases. (G) Diagnostic performance of circPRKD3/CA19-9 dual-marker panel in distinguishing PDAC patients from other pancreas-related diseases. (H) Proposed mechanism of circPRKD3-driven PDAC metastasis through MSI2 stabilization. *P < 0.05.

To evaluate clinical relevance of the circPRKD3–MSI2 regulatory axis in PDAC, we measured circPRKD3 expression by RT-qPCR and MSI2 protein levels by immunoblot analysis in 18 pairs of PDAC tumors and adjacent normal tissues. The results revealed a close positive correlation between circPRKD3 abundance and MSI2 protein expression (Fig. 8A and B and Fig. S7A and B), consistent with our experimental findings. Based on the established association between high MSI2 expression with poor clinical outcomes of PDAC patients (Fig. S4B), we investigated circPRKD3’s prognostic potential. Patients with high circPRKD3 expression (above the median expression threshold) showed more aggressive clinicopathological features, including larger tumor size and increased lymph node metastasis (Table S3). Most importantly, Kaplan–Meier survival analysis showed that elevated circPRKD3 expression was associated with markedly worse overall survival of PDAC patients (Fig. 8C), suggesting its potential as a prognostic biomarker in PDAC.

The inherent stability of circRNAs renders them excellent liquid biopsy biomarkers. Our analysis of serum samples demonstrated markedly elevated circPRKD3 in PDAC patients versus healthy controls and individuals with other pancreas-related diseases, such as pancreatic pseudocyst and chronic pancreatitis (Fig. 8D), highlighting its potential as a novel noninvasive diagnostic indicator. While conventional serum biomarkers (CA19-9, CEA, and CA125) showed suboptimal diagnostic accuracy for PDAC (Table S4), the addition of circPRKD3 substantially improved diagnostic performance. Receiver operating characteristic (ROC) analysis revealed that combining circPRKD3 with all 3 conventional biomarkers yielded superior diagnostic efficacy (area under the curve [AUC] = 0.919) compared to the conventional panel alone (AUC = 0.901) (Fig. 8E and F). Most importantly, a circPRKD3/CA19-9 dual-marker panel achieved the optimal diagnostic discrimination (AUC = 0.921; Fig. 8G), outperforming both individual markers and other combinations (Fig. S7C). Therefore, these findings indicate circPRKD3 as a promising liquid biopsy marker for PDAC detection.

## Discussion

Dysregulated circRNAs are increasingly recognized as key players in PDAC pathogenesis, exemplified by circFOXK2 that is overexpressed in 63% of PDAC cases (53/84 specimens) and drives oncogenesis through YBX1/hnRNP K-mediated transcriptional activation [36]. In this study, we identified circPRKD3 as a substantially up-regulated circRNA in PDAC tumors, exhibiting predominant cytoplasmic localization (Fig. 1). Through comprehensive gain- and loss-of-function approaches, we demonstrated that circPRKD3 functions as a master regulator of PDAC metastasis (Figs. 2 and 3). Intriguingly, circPRKD3 displays striking context-dependent functionality. For instance, Liu et al. [37] showed that circPRKD3 enhances osteogenesis in stretched periodontal ligament stem cells. Conversely, Li et al. [38] reported that circPRKD3 promotes PM_2.5_-induced pulmonary inflammation via modulating the hsa-miR-936/AKT3 axis and the PI3K (phosphatidylinositol ​3​-kinase)/AKT signaling cascade. Additionally, in glioblastoma models, tumor-derived exosomal circPRKD3 suppresses malignant progression by inhibiting STAT3 (signal transducer and activator of transcription 3) signaling and remodeling the immunosuppressive tumor microenvironment [39]. These findings demonstrate circPRKD3’s context-dependent regulatory capacity, exerting distinct biological effects across different tissue types.

Extensive investigations establish that circRNAs function as essential regulators of protein homeostasis through formation of specific ribonucleoprotein complexes. For example, circIGF1R promotes cardiac repair by interacting with DDX5 to enhance its stability and activate β-catenin signaling post-ischemia [40]. In pancreatic cancer, circCGNL1 enhances the interaction of the phosphatase NUDT4 and histone deacetylase 4 (HDAC4), promoting HDAC4 dephosphorylation and nuclear translocation to modulate gene expression [41]. In this study, we identified a direct and sequence-specific interaction between circPRKD3 and MSI2 mediated by conserved “UAGU” motifs (Figs. 4 and 5). Genetic disruption of this interaction via MSI2-binding-deficient mutants abolished circPRKD3’s pro-metastatic effects in both cellular and animal models (Fig. 5F to I), demonstrating the essential role of MSI2 binding in circPRKD3’s functions. Mechanistic analysis showed that while circPRKD3 minimally affected classical EMT markers, it dramatically regulated focal adhesion components (*FLNA*, *ITGB1*, *LAMB3*, and *PXN* mRNAs) in an MSI2-dependent manner (Fig. S4). These findings align with genomic analysis identifying focal adhesion factors as primary MSI2 targets [33], despite its reported involvement in TGF-β-induced EMT in NSCLC [19]. Collectively, these results reveal a distinct metastasis pathway in which the circPRKD3–MSI2 axis promotes PDAC metastasis through focal adhesion remodeling.

RBPs serve as master regulators of post-transcriptional gene expression, with their dysregulation being intimately linked to oncogenesis. For instance, MSI2 is markedly up-regulated across multiple malignancies including PDAC [21–23] and plays crucial roles in tumor initiation and progression through regulation of core oncogenic pathways [15]. In both genetically engineered mouse models and patient-derived xenografts, MSI2 has been shown to drive malignant progression from pancreatic intraepithelial neoplasia to invasive adenocarcinoma [22]. Moreover, single-cell transcriptomic profiling uncovers MSI2^+^ cells as multipotent precursors for diverse pancreatic cancer subtypes [23]. While KLF4-mediated transcriptional regulation contributes to MSI2 overexpression in PDAC [21], our study uncovers a novel post-translational regulatory mechanism wherein circPRKD3 binding competitively inhibits β-TRCP-mediated MSI2 ubiquitination, thereby stabilizing MSI2 to promote metastatic progression (Figs. 6 and 7). Notably, although MSI2 regulates tumor growth in various cancers [16,21,42], our findings demonstrate that the circPRKD3–MSI2 axis specifically enhances metastatic capacity without affecting proliferation in PDAC. This functional specialization mirrors observations in NSCLC, where MSI2 promotes TGF-β-driven metastasis independently of proliferative effects [19]. We propose that in KRAS-driven PDAC, characterized by constitutive proliferative signaling, circPRKD3-mediated MSI2 stabilization may preferentially enhance metastatic potential while exerting minimal effects on cell proliferation. This hypothesis will be systematically investigated in future studies.

CircRNAs have emerged as highly promising cancer biomarkers due to their exceptional stability and disease-specific expression patterns. Their abundance in biofluids makes them particularly attractive for liquid biopsy applications. For example, F-circEA, an EML4-ALK fusion-derived circRNA, has been characterized as a noninvasive biomarker in NSCLC to monitor the fusion gene [9]. Recently, Xu et al. [43] identified that the 5-circRNA plasma signature accurately discriminates PDAC patients from healthy controls and differentiates disease stages. In this study, we demonstrated that serum circPRKD3 levels were substantially elevated in PDAC patients compared to healthy individuals and patients with other pancreatic diseases (Fig. 8D). The diagnostic potential of circPRKD3 was further evidenced by its ability to enhance the performance of conventional biomarkers (CA19-9, CEA, and CA125), with combined panels achieving superior AUC values (0.919 vs. 0.901; Fig. 8E and F). Notably, the circPRKD3/CA919 dual-marker panel showed optimal diagnostic accuracy (AUC = 0.921; Fig. 8G). Clinically, elevated tumor circPRKD3 expression correlates with poorer overall survival of PDAC patients (Fig. 8C). Collectively, these findings establish circPRKD3 as a promising diagnostic and prognostic biomarker for PDAC.

In summary, this study identifies circPRKD3 as an oncogenic circRNA that promotes PDAC metastasis through direct interaction with MSI2, thereby inhibiting β-TRCP-mediated ubiquitination and subsequent proteasomal degradation (Fig. 8H). Furthermore, circPRKD3 exhibits clinical potential as a diagnostic biomarker in liquid biopsies and an independent prognostic indicator for PDAC patients.

## Materials and Methods

### Patient samples

Human specimens and associated clinical data were collected from West China Hospital of Sichuan University following approval by the Institutional Review Board (Approval #2021-1189). Written informed consents were obtained from participants or their legal representatives. Complete clinicopathological information of the study cohort is listed in Table S3.

### Cell culture

PDAC cell line PANC-1 and BxPC-3, along with human embryonic kidney cell line HEK239T, were cultured in Dulbecco’s Modified Eagle Medium (DMEM) (Gibco), while CFPAC-1, MIA PaCa2, AsPC-1, Capan-1, and Capan-2, as well as the immortalized human pancreatic duct-derived epithelial cell line HPNE, were maintained in DMEM/Ham’s F12 nutrient mixture (Gibco). All cell lines were incubated at 37 °C in a humidified 5% CO_2_ atmosphere. The culture medium was supplemented with 10% heat-inactivated fetal bovine serum (FBS, PAN-Biotech, #ST30-3302) and 1% penicillin/streptomycin (Biosharp, #P1400-100) to maintain cell viability and prevent bacterial contamination.

### Plasmid construction

The circPRKD3 expression vector was generated by cloning the exon 2 of human *PRKD3* gene into the pLacases2 vector as previously described [44]. For MSI2 expression constructs, full-length or truncated coding sequences with N-terminal Flag-tags were cloned into pCDH-CMV-MCS-EF1-Puro vector (System Biosciences). The Linear transcript mutant and the MSI2-binding-deficent mutant of circPRKD3 expression vector, along with Flag-MSI2 K22A and R100A mutants, were generated by the QuikChange II Site-Directed Mutagenesis Kit (Agilent Technologies). For gene knockdown, shRNAs targeting circPRKD3, MSI2, β-TRCP, HUWEI, or DBC2 were generated in the pLKO.1 vector. All primer sequences are provided in Table S5.

### Reverse transcription quantitative polymerase chain reaction

Total RNA was extracted using RNAiso Plus reagent (Takara, #9109) according to the manufacturer’s protocol. cDNA was synthesized from 1 μg of RNA using the PrimeScript RT reagent kit with gDNA eraser (TaKaRa, #RR047A). Quantitative PCR amplification was performed using TB Green Premix Ex Taq II (TaKaRa, #RR820A) on a QuantStudio 6 platform (Applied Biosystems), with cycle threshold values normalized to U6 small nuclear RNA or *TUBA1B* mRNA and relative quantification determined by the 2^−ΔΔCt^ method. For RT-PCR, we designed 3 distinct primer sets: Divergent and convergent primers within the exon 2 of *PRKD3* mRNA to detect circPRKD3 and total transcripts, respectively; Endogenous *PRKD3* mRNA primers binding outside of the exon 2 to specifically amplify endogenous mRNA without detecting lentivirus-derived linear transcripts; and Linear transcript-specific primers with the forward primer in the upstream flanking region of circPRKD3 lentiviral vector and the reverse primer within the linear circPRKD3 sequence to exclusively amplify the lentivirus-produced transcript. All primer sequences are listed in Table S6.

### Transcriptome RNA-sequencing

Total RNA was extracted from tissue samples or cultured cells and subjected to Ribo-Zero rRNA-depleted RNA sequencing. CircRNAs were identified using CIRC2 algorithm and annotated against the circBase reference database. Differential expression analysis of circRNAs in PDAC patient samples was performed with thresholds of |Log_2_ fold change| ≥1.0 and *P* value <0.05. Differential expression analysis of genes in cultured PDAC cells was analyzed using the R package DESeq2 and defined as |Log_2_ fold change| ≥0.585 and *P* value <0.05. GO enrichment was analyzed using R with the clusterProfiler package (version 4.3.1).

### RNase R digestion and actinomycin D treatment

Total RNA (10 μg) was treated with RNase R (10 U; Epicentre Technologies #RNR07250) at 37 °C for 10 min to selectively degrade linear RNAs while preserving circular transcripts. Following enzymatic digestion, circRNAs were reverse transcribed using the PrimeScript RT reagent kit and amplified with 10×Taq PCR mix. For transcriptional inhibition studies, PDAC cells (2 × 10^5^ cells/well) were seeded in 6-well plates and treated with 2 μg/ml actinomycin D prior to RNA extraction and subsequent RT-qPCR analysis.

### Subcellular fractionation

Cells were lysed in the hypotonic buffer (25 mM Tris-HCl, pH 7.4, 5 mM KCl, 1.5 mM MgCl_2_, and 0.5% NP-40) on ice for 10 min, followed by centrifugation at 5,000 ×*g* for 5 min at 4 °C. The supernatants were collected as the cytosolic fraction, while pellets were washed once with the hypotonic buffer and subsequently lysed in RIPA buffer (Beyotime, #P0013B) on ice for 15 min. Nuclear fractions were obtained by centrifugation at 16,000 ×*g* for 10 min at 4 °C. Fractionation efficiency was verified by immunoblot analysis using GAPDH (cytosolic marker) and Lamin A/C (nuclear marker).

### Immunoblot analysis

Cell and tissue lysates were prepared using RIPA buffer (Beyotime, #P0013B) supplemented with complete protease inhibitors (Roche, #PPC1010), and their protein concentrations were determined by the BCA assay kit (Beyotime, #P0009). Proteins were resolved on sodium dodecyl sulfate (SDS)-polyacrylamide gels and transferred onto PVDF (polyvinylidene fluoride) membranes (GE Healthcare, #10600021). Membranes were blocked with 5% nonfat milk and incubated with primary antibodies at 4 °C overnight, followed by incubation with secondary antibodies at room temperature for 1 h. Protein detection was performed using SuperSignal West Dura Extended Duration Substrate Enhanced (Thermo Fisher Scientific, #34076) on a ChemiDoc XRS^+^ System (Bio-Rad). The following antibodies were used: Lamin A/C (Abways, #CY5222; 1:1,000), GAPDH (Cell Signaling Technology, #3683; 1:5,000), MSI2 (Proteintech, #10770; 1:1,000), PABPC1 (Proteintech, #10970; 1:1,000), Flag (Proteintech, #66008; 1:1,000), Tubulin (Abways, #AB0049; 1:5,000), Ubiquitin (Cell Signaling Technology, #3936; 1:1,000), β-TRCP (Cell Signaling Technology, #4394; 1:1,000), E-cadherin (Cell Signaling Technology, #3195; 1:1,000), N-cadherin (Cell Signaling Technology, #4061; 1:1,000), and Vimentin (Cell Signaling Technology, #3932; 1:1,000). Uncropped immunoblot images are provided in the Supplementary Materials.

### Cell migration and invasion assays

For migration assay, PANC-1 (3 × 10^4^ cells), CFPAC-1 (5 × 10^4^ cells), or Capan-2 (7.5 × 10^4^ cells) cells were suspended in serum-free RPMI-1640 medium supplemented with 5% bovine serum albumin (BSA) and seeded into the upper chamber of a Transwell insert (Falcon, #FAL-353097). The lower compartment was filled with 500 μl of RMPI-1640 medium containing 10% FBS as a chemoattractant. For invasion assessment, the upper chamber surface was precoated with 60 μl of Matrigel (Corning, #356231) diluted 1:8 in RPMI-1640 medium and allowed to polymerize. Subsequently, PANC-1 (5 × 10^4^ cells), CFPAC-1 (7.5 × 10^4^ cells), or Capan-2 (1 × 10^5^ cells) cells were suspended in serum-free RPMI-1640 medium containing 5% BSA and plated onto the Matrigel-coated inert. Following a 36-h incubation period for migration and invasion assays, cells that had traversed the membrane were fixed with 4% paraformaldehyde for 15 min and stained with 0.5% crystal violet solution for 30 min. In the wound healing assay, cell monolayers were prepared by seeding cells in 6-well plates and culturing them to ~90% confluence. A linear scratch was introduced into the cell monolayer using a sterile pipette tip. After washing to remove dislodged cells, the plates were cultured for an additional 24 or 48 h. Images of the wound gaps were captured at 0, 24, and 48 h. The percentage of wound closure was calculated using the formula: Wound closure (%) = (1 − width at 24 or 48 h/width at 0 h) × 100%.

### Cell proliferation and colony formation assays

For MTT cell proliferation assay, PANC-1 (2,000 cells/well), CFPAC-1 (2,500 cells/well), and Capan-2 (5,000 cells/well) cells were seeded into 96-well plates and cultured for the indicated time intervals. On days 1, 3 and 5, 10 μl of MTT solution (Sigma, #M5655) was added to each well, followed by incubation for 4 h at 37 °C. After solubilizing the formazan crystals with 150 μl of DMSO, absorbance was measured at 490 nm using a BioTek microplate reader. For colony formation analysis, PANC-1 (2,000 cells/well), CFPAC-1 (2,000 cells/well), and Capan-2 (4,000 cells /well) cells were plated in 6-well plates and cultured for 14 days. Following incubation, colonies were fixed with 4% paraformaldehyde for 15 min and stained with 0.5% crystal violet for 30 min.

### Fluorescence in situ hybridization

RNA FISH was performed using the Ribo Fluorescent In Situ Hybridization Kit (RiboBio, China) following the manufacturer’s protocols. Cy3-labeled probes targeting circPRKD3 junction sequences, U6 snRNA, or 18S rRNA are listed in Table S7. Briefly, PANC-1 or CFPAC-1 cells grown on coverslips were fixed with 4% paraformaldehyde for 10 min, permeabilized with 0.5% Triton X-100 for 5 min, and hybridized with 20 nM probes in a humidified chamber at 37 °C overnight. Nuclei were counterstained with 4′,6-diamidino-2-phenylindole (DAPI) solution for 30 min, and images were acquired using an OLYMPUS FV1000 confocal microscope.

### In vitro cyclization of circRNA

Linear RNA transcripts were synthesized using the MAXIscript T7/T3 Transcription Kit (Thermo Fisher Scientific) according to the manufacturer’s instructions. The resulting RNAs were then labeled with biotin using the Biotin RNA Labeling Mix (Roche) following the recommended protocol. For annealing, biotin-labeled linear RNAs were mixed with DNA splints at a 1: 1.5 molar ratio in annealing buffer (10 mM Tris-HCl, pH 7.5, and 100 mM NaCl) and heated at 95 °C for 5 min, followed by gradual cooling to room temperature. RNA circularization was performed by incubating the annealed products with T4 DNA ligase (NEB) at 16 °C overnight. To purify circRNAs, the reaction products were sequentially treated with RNase R (Epicentre) and DNase I (Thermo Fisher Scientific) for 30 min at 37 °C to remove linear RNA and DNA splints, respectively. The circRNAs were then extracted by phenol-chloroform and precipitated with ethanol. All primers and DNA splints used for in vitro cyclization of circPRKD3 are listed in Table S7.

### RNA pull-down assays

For DNA probe-based RNA pull-down assays, PANC-1 cells were transfected with 400 pmol of biotinylated DNA probes complementary (antisense, AS) or identical (sense, S) to the circPRKD3 back-splicing junction. After 24 h of transfection, cells were lysed in ice-cold lysis buffer (20 mM Tris-HCl, pH 7.5, 140 mM NaCl, 1.5 mM MgCl_2_, 0.5% NP-40, and 1 mM dithiothreitol) supplemented with protease inhibitor cocktail (Roche) and RNase inhibitors (Thermo Fisher Scientific). The resulting lysates were incubated with prewashed streptavidin-conjugated magnetic beads at 4 °C for 4 h with constant rotation. Following 3 washes with high-salt washing buffer (20 mM Tris-HCl, pH 7.5, 1 mM EDTA, and 300 mM NaCl), the bead-bound complexes were divided for downstream analyses: one-third was subjected to RNA isolation and subsequent RT-qPCR quantification, while the remaining two-thirds were boiled in 50 μl of 1× SDS loading buffer for immunoblot analysis.

For pull-down assays using biotin-labeled RNA probes, 100 pmol of biotinylated RNA probes was incubated with pre-equilibrated streptavidin magnetic beads at 4 °C for 1 h with constant rotation. Next, the beads were washed once with high-stringency buffer (20 mM Tris-HCl, pH 7.5, 1 mM EDTA, and 450 mM NaCl). The RNA-conjugated beads were then incubated with either PANC-1 cell lysates or recombinant MSI2 protein at 4 °C for 4 h. After extensive washing, the captured protein–RNA complexes were eluted by boiling in 50 μl of 1× SDS loading buffer for 5 min, followed by immediate cooling on ice prior to SDS-PAGE and immunoblot analysis.

To perform pull-down assays with in vitro cyclized circRNA, sense strand (S) or antisense strand (AS) of circPRKD3 (2 μg) were first denatured at 95 °C for 5 min, followed by gradual cooling to room temperature to allow proper secondary structure formation. The renatured circRNAs were then incubated with PANC-1 cell lysates at 4 °C for 4 h. Streptavidin magnetic beads were added to the RNA–protein complex and incubated at 4 °C for an additional hour. After magnetic separation, the bead-bound complexes were washed 3 times with high-stringency washing buffer and eluted by boiling in 50 μl of 1× SDS loading buffer. All primer and probe sequences used in these pull-down assays are provided in Table S7.

### IP assays

Cells were lysed in ice-cold IP buffer (20 mM Tris-HCl, pH 7.5, 140 mM NaCl, 1.5 mM MgCl_2_, 0.5% NP-40, 1 mM EDTA, and 10% glycerol) containing protease inhibitors (Roche) and RNase inhibitors (Thermo Fisher Scientific) at 4 °C for 15 min, followed by Dounce homogenization. After centrifugation at 16,000 ×*g* for 15 min at 4 °C, the resulting supernatants were immunoprecipitated with 2 μg of anti-MSI2 antibody (Proteintech, #10770) or control IgG overnight at 4 °C with rotation. Protein complexes were captured using Protein A/G Agarose beads (Millipore, #IP10) for 2 h at 4 °C. The bead-bound proteins were analyzed by immunoblot analysis, while the co-precipitated RNAs were purified for RT-PCR analysis.

For Flag-tagged protein studies, cells were transfected with either Flag-tagged expression constructs or empty vector. Following lysis, clarified supernatants were incubated with ANTI-FLAG M2 Affinity Gel (Sigma, #A2220) at 4 °C for 4 h. The bound proteins were analyzed by immunoblot analysis, while co-precipitated RNAs were isolated for RT-qPCR analysis. For ubiquitination assays, PANC-1 or CFPAC-1 cells were transfected with HA-ubiquitin expression vectors for 48 h prior to lysis. Ubiquitinated MSI2 was immunoprecipitated and detected by anti-HA antibodies.

### Recombinant protein production and purification

*Escherichia coli* BL21(DE3) were transformed with MSI2 expression plasmids, and protein expression was induced with 0.1 mM IPTG at 16 °C for 24 h. Bacterial pellets were lysed by sonication and recombinant GST-tagged MSI2 was purified using GST affinity chromatography with elution buffer (20 mM Tris-HCl, pH 8.0, 150 mM NaCl, and 1 mM DTT). The eluate was concentrated and stored at −80 °C in aliquots until further analysis.

### Electrophoretic mobility shift assays

RNA–protein interactions were characterized using a Chemiluminescent EMSA Kit (Beyotime, #GS009) according to the manufacturer’s protocol. Briefly, purified MSI2 protein was incubated with biotinylated RNA probes in binding buffer at 25 °C for 1 h. The reaction mixture was then resolved on 5% native polyacrylamide gel at 60 V for 2 h. Nucleic acids were transferred to positively charged nylon membranes and ultraviolet crosslinked at 254 nm (200 mJ/cm^2^) for 2 min. Membranes were blocked with 5% BSA for 30 min at room temperature, then probed with horseradish peroxidase (HRP)-conjugated streptavidin for 30 min at room temperature. Complexes were detected using visualized using SuperSignal West Pico Chemiluminescent Substrate (Thermo Fisher Scientific, #34076) and imaged on a ChemiDoc XRS^+^ System (Bio-Rad). All probe sequences used for EMSAs are listed in Table S7.

### Animal models

All animal experiments were conducted in accordance with the approved protocols by the Institutional Animal Care and Use Committee of West China Hospital, Sichuan University (Approval #20220307005). Four-week-old female NSIG mice were obtained from HFK Bioscience Co., Ltd. (Beijing, China) and randomly allocated into experimental groups (*n* = 5 per group). For the pulmonary metastasis model, NSIG mice were intravenously injected via the tail vein with 1 × 10^6^ luciferase-labeled CFPAC-1 cells. For the hepatic metastasis model, NSIG mice received an intrasplenic injection of 1 × 10^6^ luciferase-labeled PANC-1 cells. The spleen was surgically excised 5 min post-injection. Four weeks after tumor cell inoculation, mice were intraperitoneally injected with 100 μl of D-luciferin (15 mg/ml, Promega) and anesthetized for bioluminescence imaging. Metastatic burden was quantified using the IVIS SpectrumCT imaging system (Revvity), and signal analysis was performed with Living Image 4.8.2 software (Caliper Life Sciences). Upon termination of the experiment, mice were euthanized, and lung/liver tissues were harvested and fixed in 4% formalin for further histopathological examination.

### IHC staining

Tissue specimens were embedded in paraffin and sectioned into 4 μm thickness. Sections were deparaffinized in xylene and rehydrated through a graded ethanol series (100%, 95%, 85%, and 75%). Antigen retrieval was performed by heat-mediated epitope unmasking in 20 mM sodium citrate buffer (pH 6.0). To quench endogenous peroxidase activity, sections were incubated with 1% H_2_O_2_ for 15 min at room temperature. To minimize nonspecific antibody binding, sections were blocked with 5% normal goat serum for 30 min. Following incubation overnight at 4 °C with MSI2 antibody (Proteintech, #10770, 1:400 dilution) or Nucleolin antibody (Abcam, #ab136649, 1:200 dilution), sections were treated with an HRP-conjugated secondary antibody for 1 h at room temperature. Finally, immunoreactivity was visualized using diaminobenzidine tetrahydrochloride (DAB) as the chromogenic substrate. Sections were counterstained with H&E for 5 min to assess histomorphology.

### Statistical analysis

All statistical analyses were performed with GraphPad Prism 8.0 software (GraphPad Software). Group comparisons utilized 2-tailed Student’s *t* tests or one-way analysis of variance. Quantitative data were expressed as mean ± standard deviation (SD), with statistical significance defined as *P* value < 0.05.

## Ethical Approval

The mouse procedures were reviewed and approved by the Institutional Animal Care and Use Committee of West China Hospital, Sichuan University (Approval #20220307005). The protocols for human subjects were followed with the ethical standards of the institutional committee of West China Hospital, Sichuan University (Approval #2021-1189).

## Data Availability

The raw sequence data generated in this study have been deposited in the Genome Sequence Archive (GSA) in the National Genomics Data Center (China National Center for Bioinformation/Beijing Institute of Genomics, Chinese Academy of Sciences) under accession numbers HRA010581 and HRA010566. These datasets are available through the GSA-Human repository (https://ngdc.cncb.ac.cn/gsa-human). Mass spectrometry proteomics data have been submitted to the ProteomeXchange Consortium (https://proteomecentral.proteomexchange.org) via the iProX repository with the dataset identifier PXD061484.

## Supplementary Materials

Supplementary 1 Figs. S1 to S7 Tables S1 to S7 Uncropped immunoblot images
