# Supplementary material for: A Circular RNA Promotes Tumor Metastasis through Stabilizing MSI2 Protein in Pancreatic Ductal Adenocarcinoma
Source: Research (Wash D C). 2025 Oct 3;8:0918. doi: 10.34133/research.0918 (PMC12491784; doi:10.34133/research.0918)

SUPPORTING INFORMATION

A circular RNA promotes tumor metastasis through stabilizing MSI2 protein in pancreatic ductal adenocarcinoma

Zhang Li[1†], Lanyang Gao[1†], Yang Yang[2†], Yue Ming[1], Wenrong Liu[1], Tingting Zhang[1], Zixia Ye[1], Fuyan Xu[1], Juan He[1], Jiao Li[1], Jiawei Guo[1], Xiaojuan Yang[1], Qing Zhu[2*], Yong Peng[1, 3*]

[1] Center for Molecular Oncology, Frontiers Science Center for Disease-related Molecular Network, State Key Laboratory of Biotherapy and Cancer Center, State Key Laboratory of Respiratory Health and Multimorbidity, West China Hospital, Sichuan University, Chengdu 610041, China. [2] Division of Abdominal Tumor Multimodality Treatment, Cancer Center, Department of General Surgery, West China Hospital, Sichuan University, Chengdu 610041, China. [3] Frontiers Medical Center, Tianfu Jincheng Laboratory, Chengdu 610212, China.

[*] Address correspondence to: yongpeng@scu.edu.cn (Y.P.); newzhuqing1972@163.com (Q.Z.).

[†] These authors contributed equally to this article.

Running title: circPRKD3 promotes PDAC metastasis.

This PDF file includes:

Figure S1-S7

Table S1-S7

Uncropped Immunoblot Images

Supplementary Figures

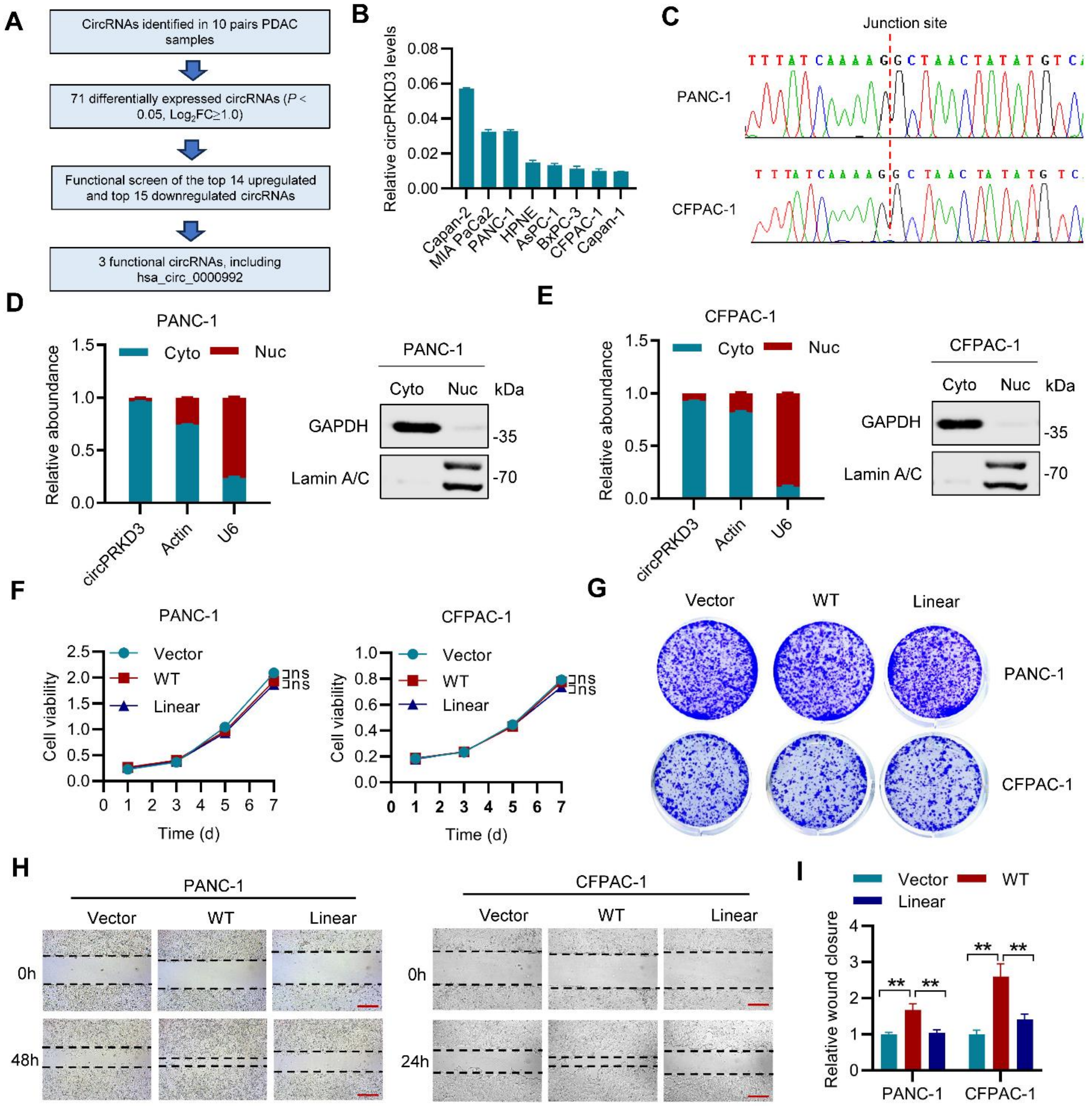

Figure S1. Characterization of circPRKD3 overexpression in PDAC cells.

A, Workflow for circRNA profiling in 10 matched PDAC tumor-normal pairs. B, Endogenous circPRKD3 expression across PDAC cell lines measured by RT-qPCR. C, Sanger sequencing confirmation of back-splice junctions of ectopically expressed circPRKD3. D-E, Subcellular localization of ectopically expressed circPRKD3. Cellular fractionations were prepared from circPRKD3-overexpressing PANC-1 (D) and CFPAC-1 (E) cells, followed by RT-qPCR analysis to measure circPRKD3 levels. *β-actin* mRNA and U6 snRNA served as cytoplasmic (Cyto) and nuclear (Nuc) markers. Immunoblot controls: GAPDH for cytoplasm, Lamin A/C for nucleus). F-G, Effects of wild-

type (WT) circPRKD3 or the linear mutant (Linear) on cell proliferation (F) and colony formation (G) in PANC-1 and CFPAC-1 cells. H-I, Representative images (H) and their quantifications (I) showing the effects of WT circPRKD3 or the linear mutant (Linear) on wound healing in PANC-1 and CFPAC-1 cells. Scale bar= 200 μm. ***P* <0.01, ns= not significant.

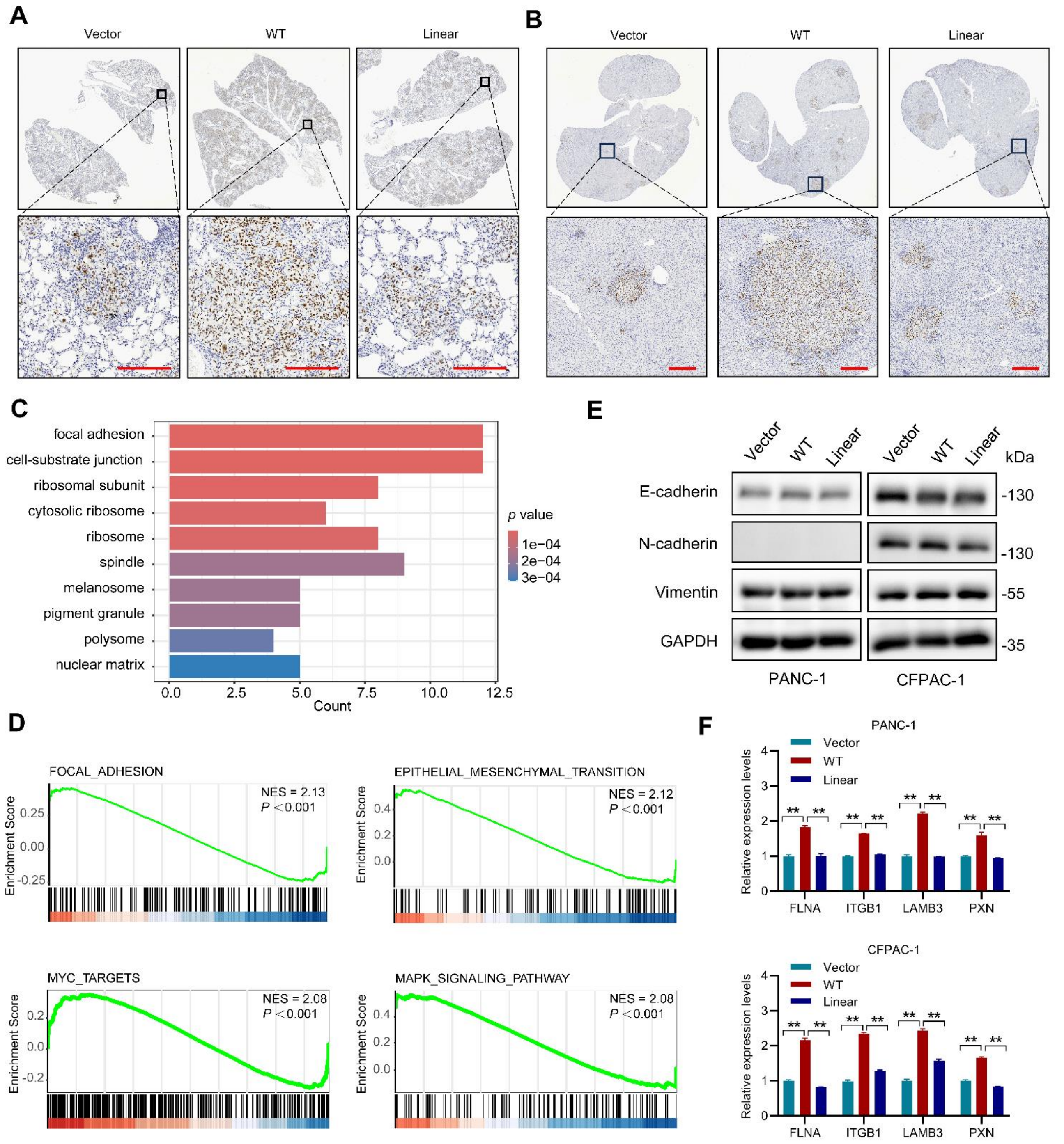

Figure S2. Functional consequences of circPRKD3 overexpression in PDAC cells.

A-B, Metastasis validation by nucleolin IHC staining in lung (A) and liver (B) sections from NSIG mice bearing PDAC xenografts (Scale bars= 200 μm). C, Top enriched GO terms among differentially expressed genes in circPRKD3-overexpressing PANC-1 cells. D, GSEA analysis identifying hallmark pathways associated with circPRKD3 overexpression in PANC-1 cells. E, Immunoblot analysis confirming that circPRKD3 did not alter EMT

marker expression. F, RT-qPCR analysis showing circPRKD3-mediated upregulation of focal adhesion components (*FLNA*, *ITGB1*, *LAMB3* and *PXN* mRNAs) in PDAC cells. $^{**}P < 0.01$.

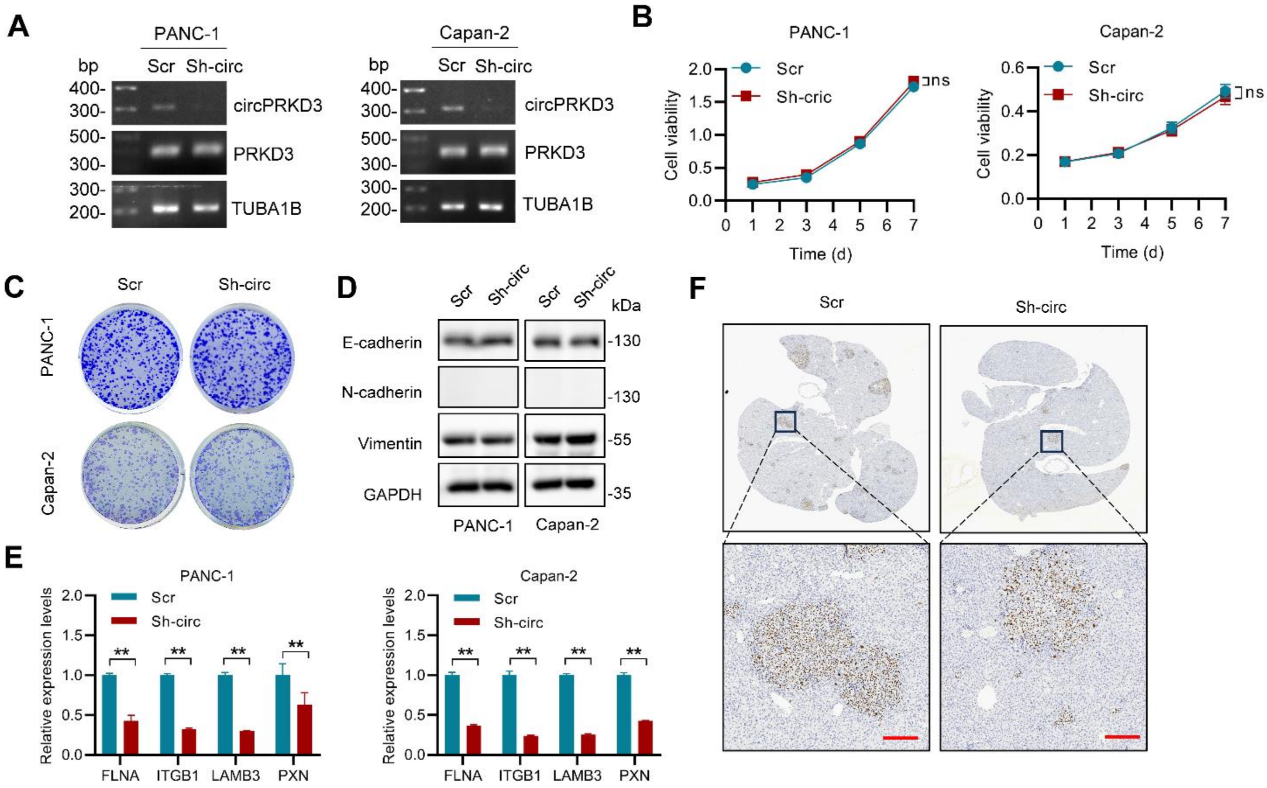

Figure S3. Functional characterization of circPRKD3 knockdown in PDAC cells.

A, CircRNA-specific knockdown validation by RT-PCR, showing selective depletion of circPRKD3 (but not *PRKD3* mRNA) in PANC-1 and Capan-2 cells. B-C, MTT assays (B) and colony formation assays (C) showing no significant effects of circPRKD3 knockdown on cell proliferation. D, Immunoblot analysis showing that circPRKD3 depletion had no obvious influence on EMT marker expression. E, RT-qPCR analysis indicating the downregulation of focal adhesion components (*FLNA*, *ITGB1*, *LAMB3* and *PXN* mRNAs) by circPRKD3 knockdown in PDAC cells. F, Representative IHC staining of nucleolin in liver metastasis of NSIG mice receiving intrasplenic injection of PANC-1 cells with/without circPRKD3 knockdown. Scale bar= 200 μm. $^{**}P$ <0.01, ns= not significant.

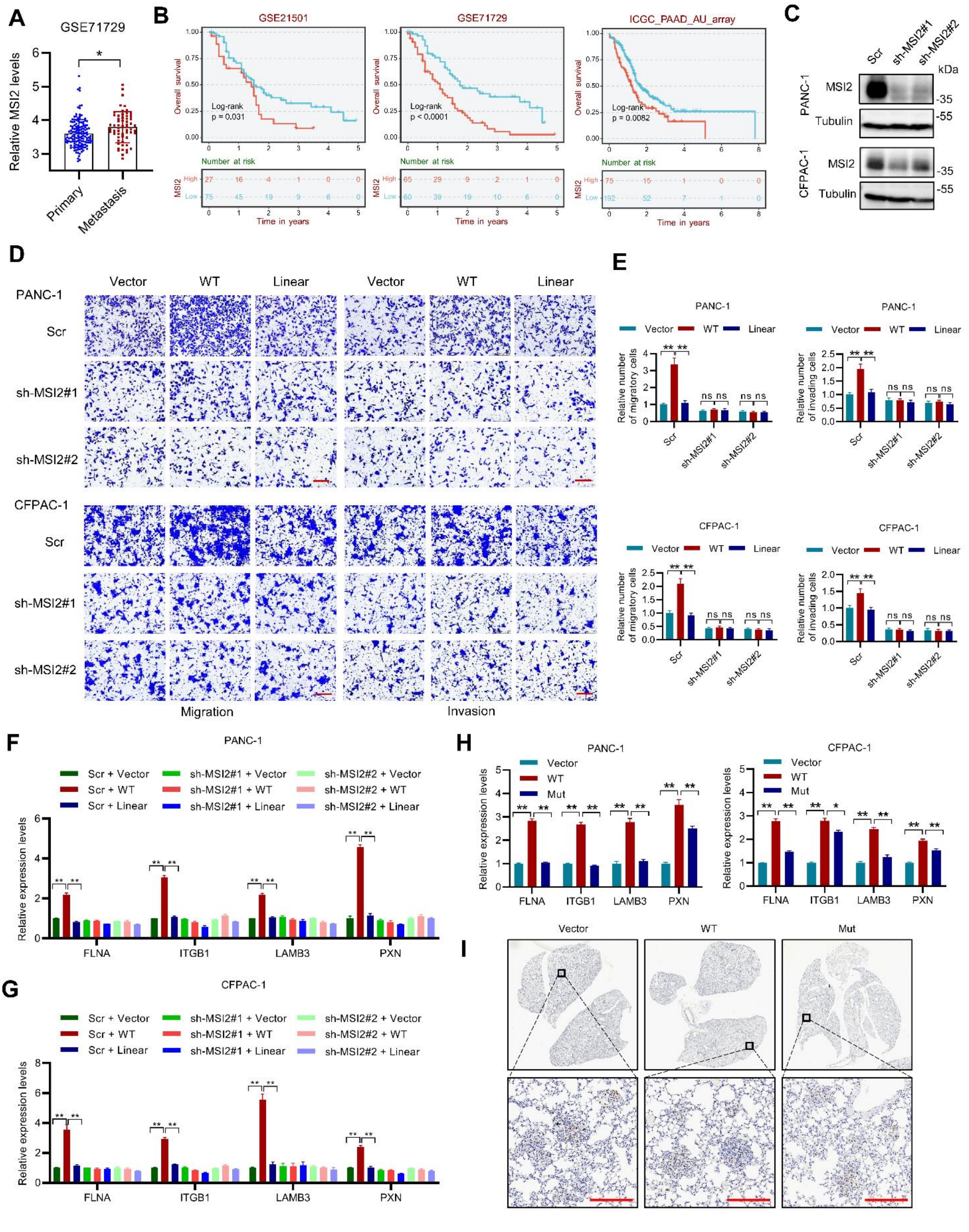

Figure S4. MSI2 mediates circPRKD3's pro-metastatic effects in PDAC.

A, MSI2 upregulation in metastatic specimens versus primary PDAC tumors. B, High MSI2 expression correlated with poor overall survival of PDAC patients across three cohorts (GSE21501, GSE71729 and

ICGC_PAAD_AU_array datasets). C, Immunoblot validation of MSI2 knockdown efficiency by two independent shRNAs (versus scrambled control, Scr) in PDAC cells. D-E, Representative images (D) and their quantification (E) of Transwell assays showing MSI2 knockdown abolishes circPRKD3-enahanced migration and invasion of PDAC cells. Scale bar = 250 μm. F-G, RT-qPCR analysis showing the MSI2-dependent regulation of focal adhesion components (*FLNA*, *ITGB1*, *LAMB3* and *PXN* mNRAs) in PANC-1 (F) and CFPAC-1 cells (G). H, RT-qPCR analysis showing the effect of MSI2-binding-deficient mutant (Mut) on the expression of focal adhesion components (*FLNA*, *ITGB1*, *LAMB3* and *PXN* mNRAs) in PDAC cells. I, Representative IHC staining of nucleolin in lung metastasis in NSIG mice intravenously injected with CFPAC-1 cells expressing empty vector (Vector), WT circPRKD3 or its MSI2-binding mutant (Mut). Scale bar = 200 μm. *P <0.05, **P <0.01, ns= not significant.

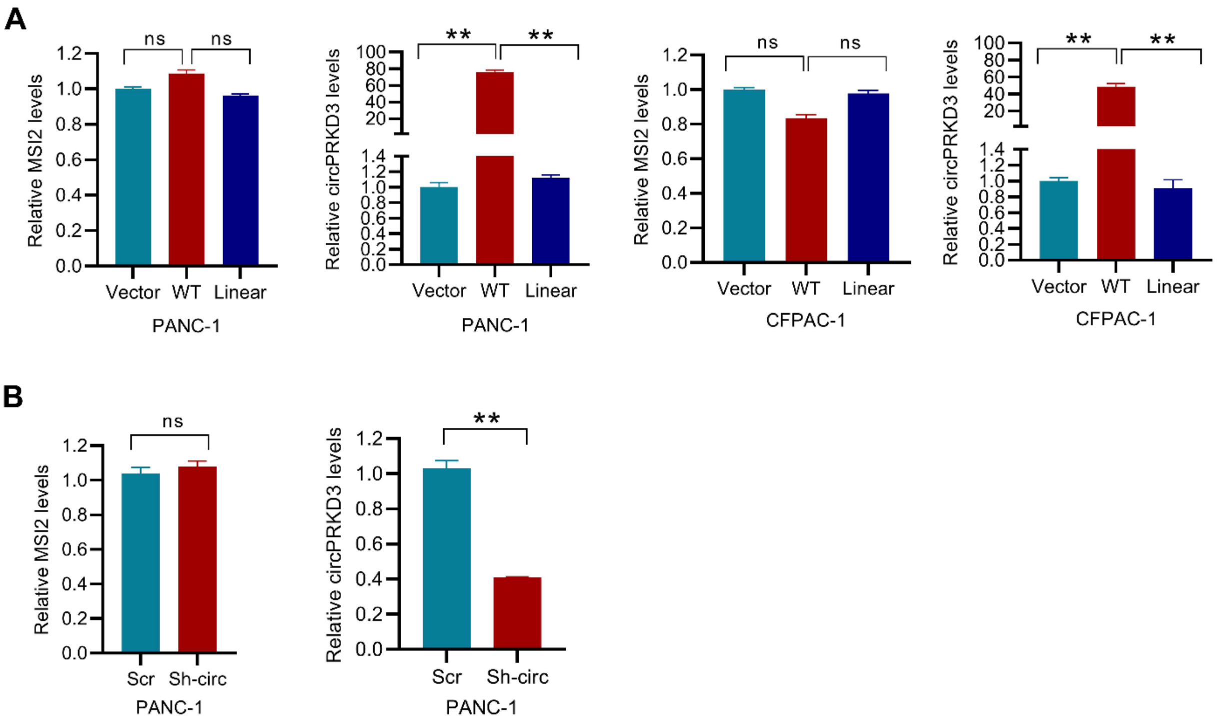

Figure S5. CircPRKD3 regulates MSI2 at the protein but not mRNA level.

A. RT-qPCR analysis showing effects of wild type (WT) circPRKD3 and the linear transcript mutant (Linear) on *MSI2* mRNA levels in PDAC cells. B. RT-qPCR analysis showing effects of circPRKD3 knockdown on *MSI2* mRNA levels in PANC-1 cells. ***P* <0.01, ns, not significant.

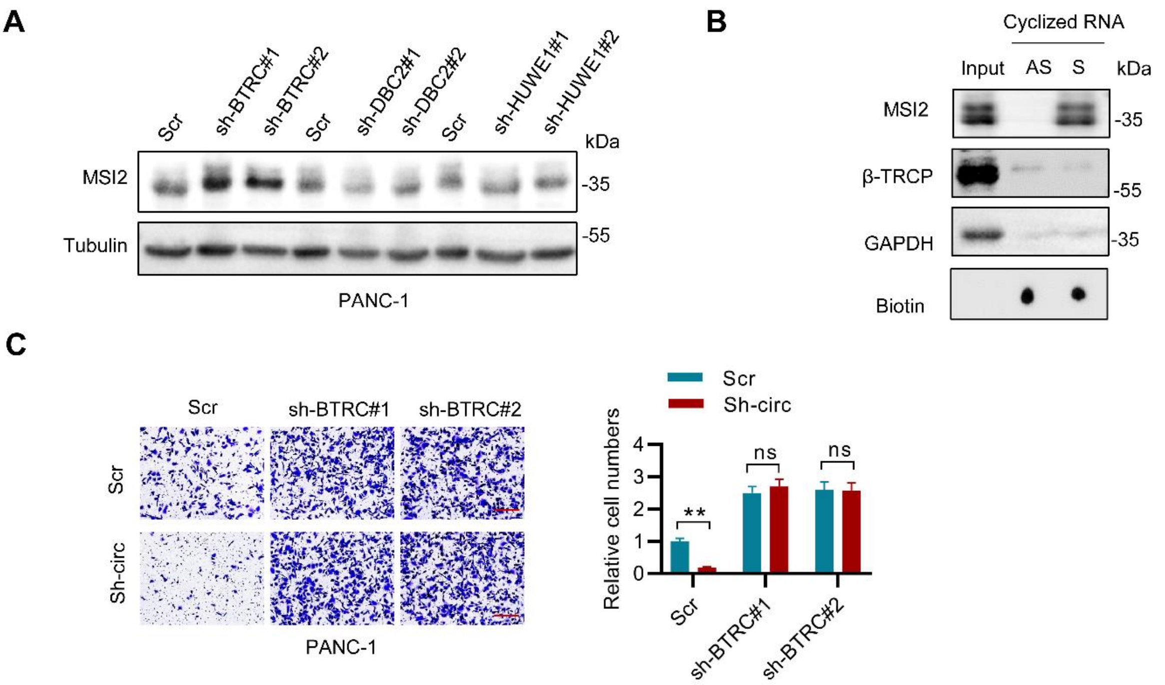

Figure S6. β-TRCP mediates the functional effects of circPRKD3 depletion in PDAC cells.

A, Immunoblot analysis showing β-TRCP knockdown (but not HUWE1 or DBC2 depletion) increased MSI2 protein levels in PANC-1 cells. B, RNA pull-down assays confirming no enrichment of β-TRCP by *in vitro* circularized circPRKD3. C, β-TRCP knockdown rescued the inhibitory effects of circPRKD3 depletion in PDAC cells, measured by Transwell migration and invasion assays. Scale bar= 250 μm. ***P* <0.01, ns, not significant.

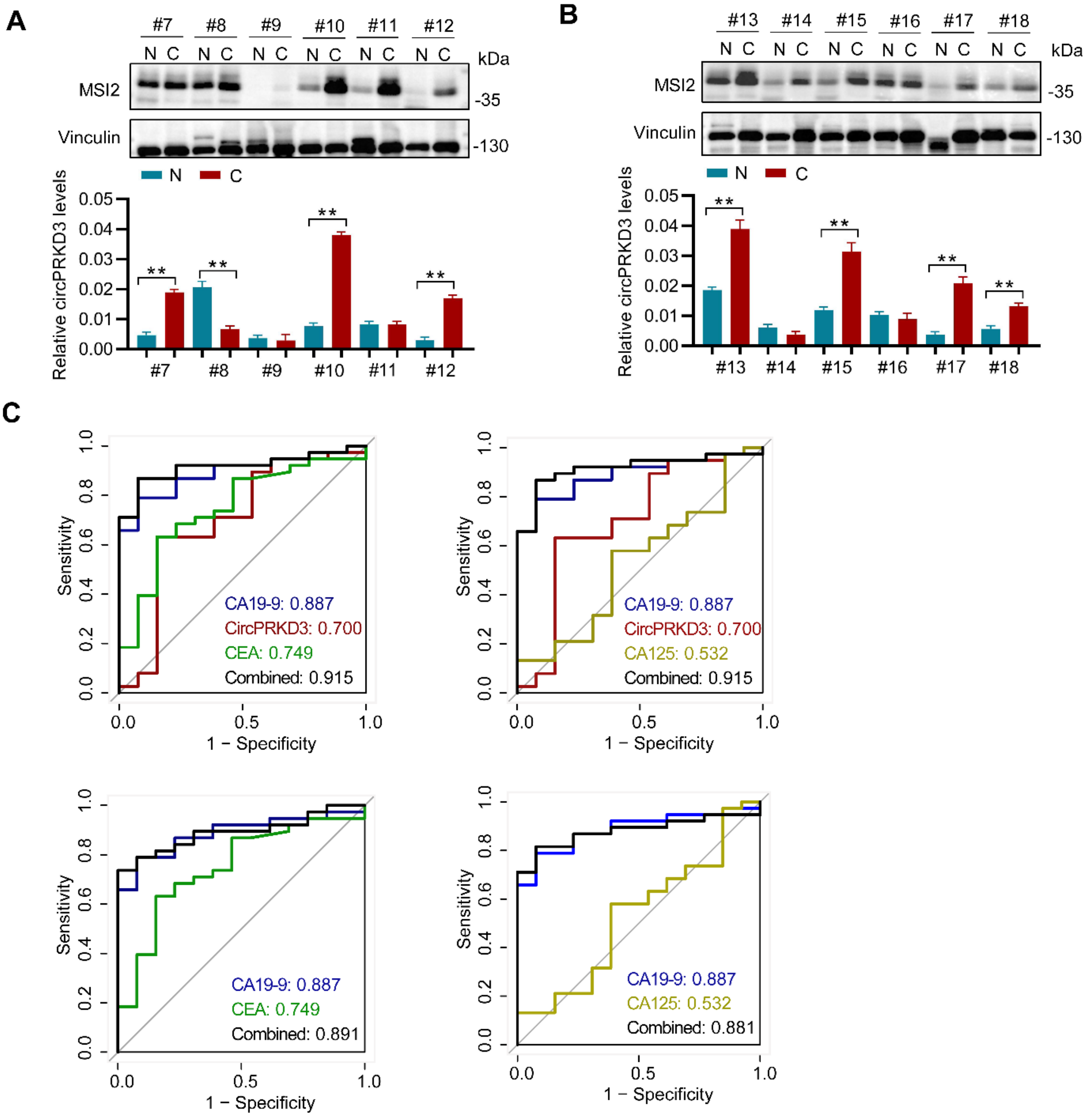

Figure S7. Clinical significance of MSI2 and circPRKD3 in PDAC.

A-B, MSI2 and circPRKD3 expression were measured by immunoblot (top) and RT-qPCR analysis (bottom) in paired PDAC tumors (C) and adjacent normal tissues (N). C, ROC analysis comparing diagnostic performance of circPRKD3, CA19-9, CEA, CA125 and combined panels for distinguishing PDAC from other pancreas-related diseases. ***P* <0.01.

Table S1. Differentially expressed circRNAs in paired PDAC tumors and adjacent normal tissues

chr	start	end	strand	host gene	circBase ID	log_2 FC	*P* value
chr12	94562928	94580249	+	PLXNC1	hsa_circ_0099504	4.639749	1.31E-16
chr1	114372213	114377061	-	PTPN22	hsa_circ_0000110	2.572277	4.15E-06
chr12	109046047	109048186	-	CORO1C	hsa_circ_0000437	2.531634	5.11E-07
chr17	20107645	20109225	+	SPECC1	hsa_circ_0000745	2.467819	6.26E-14
chr2	40655612	40657444	-	SLC8A1	hsa_circ_0000994	2.462053	1.10E-11
chr6	87925620	87928449	+	ZNF292	hsa_circ_0004058	2.018089	7.04E-07
chr3	145838898	145842016	-	PLOD2	hsa_circ_0122319	1.666772	3.58E-05
chr2	37543379	37544322	-	PRKD3	hsa_circ_0000992	1.341579	0.000651
chr2	61749745	61761038	-	XPO1	hsa_circ_0001017	1.250131	3.78E-06
chr6	47251673	47254331	-	TNFRSF21	hsa_circ_0001610	1.216386	0.002164
chr8	99718694	99719539	-	STK3	hsa_circ_0004592	1.128427	0.000973
chr17	43552465	43553092	-	PLEKHM1	hsa_circ_0044177	1.066327	0.004503
chr5	137320945	137324004	-	FAM13B	hsa_circ_0001535	1.057414	6.21E-06
chr5	122881110	122893258	+	CSNK1G3	hsa_circ_0001522	1.013671	7.94E-05
chr1	185183638	185200840	+	SWT1	hsa_circ_0004689	-1.02929	0.007122
chr10	7839009	7844817	+	ATP5C1	hsa_circ_0007292	-1.05015	4.34E-05
chr2	64778576	64780543	+	AFTPH	hsa_circ_0001021	-1.08101	0.005225
chr6	137015277	137019820	-	MAP3K5	hsa_circ_0006856	-1.08981	0.012243
chr1	104108056	104118162	+	AMY2B	hsa_circ_0000099	-1.1293	0.002218
chr14	31596990	31641328	-	HECTD1	hsa_circ_0031482	-1.15801	0.008548
chr6	145103057	145124283	+	UTRN	hsa_circ_0001648	-1.16324	0.004238
chr21	30693541	30702014	+	BACH1	hsa_circ_0001181	-1.16426	1.06E-05
chr5	179688683	179707608	-	MAPK9	hsa_circ_0001566	-1.16633	0.001184
chr6	7176887	7189555	+	RREB1	hsa_circ_0001573	-1.18051	6.36E-05
chr1	117944807	117963271	+	MAN1A2	hsa_circ_0000118	-1.18717	5.98E-05
chr5	38523520	38530768	-	LIFR	hsa_circ_0072309	-1.18841	0.000529
chr8	37727937	37735069	-	RAB11FIP1	hsa_circ_0005630	-1.19277	0.005616
chr5	95091099	95099324	+	RHOBTB3	hsa_circ_0007444	-1.20773	0.000658
chr1	117944807	117984947	+	MAN1A2	hsa_circ_0000119	-1.31682	1.36E-05
chr9	99284787	99327765	-	CDC14B	hsa_circ_0087641	-1.34599	0.00256
chr7	22330793	22357656	-	RAPGEF5	hsa_circ_0001681	-1.40681	9.98E-05
chr7	40027197	40041630	+	CDK13	hsa_circ_0001699	-1.40962	0.001602
chr2	165548730	165552346	-	COBLL1	hsa_circ_0117890	-1.49863	0.000484
chr14	32559707	32586493	+	ARHGAP5	hsa_circ_0031584	-1.5037	4.42E-05
chr4	1219147	1235307	-	CTBP1	hsa_circ_0001386	-1.51824	0.000727
chr1	225140371	225161855	+	DNAH14	hsa_circ_0016600	-1.52173	0.001617
chrX	130883333	130928494	-	RP11-453F18	hsa_circ_0001944	-1.56566	0.000912
chr1	95609446	95616975	+	TMEM56	hsa_circ_0000095	-1.60415	0.001002
chr14	31596990	31602881	-	HECTD1	hsa_circ_0002301	-1.63019	0.000251

chr	start	end	strand	host gene	circBase ID	$log_2 FC$	*P* value
chr2	207144263	207162097	+	ZDBF2	hsa_circ_0002141	-1.69451	0.002391
chr4	103225473	103236987	-	SLC39A8	hsa_circ_0002782	-1.71976	0.000347
chr1	95609446	95639445	+	TMEM56	hsa_circ_0005720	-1.7234	0.00032
chr8	17601112	17613470	-	MTUS1	hsa_circ_0083444	-1.73853	6.06E-05
chr1	118003110	118045592	+	MAN1A2	hsa_circ_0002059	-1.78993	0.000114
chr1	78097534	78107340	-	ZZZ3	hsa_circ_0114166	-1.98009	8.36E-06
chr13	96409897	96416207	+	DNAJC3	hsa_circ_0101041	-2.09306	2.89E-08
chr10	128768965	128926028	+	DOCK1	hsa_circ_0020397	-2.10923	3.58E-05
chr7	97820039	97823884	+	LMTK2	hsa_circ_0001725	-2.17149	2.27E-09
chr7	22347957	22357656	-	RAPGEF5	hsa_circ_0133954	-2.2063	3.29E-11
chr4	187627716	187630999	-	FAT1	hsa_circ_0001461	-2.23586	2.65E-07
chr14	92264128	92268765	-	TC2N	hsa_circ_0032969	-2.23644	1.15E-05
chr3	18419661	18462483	-	SATB1	hsa_circ_0064555	-2.25945	3.25E-05
chr18	42529845	42533305	+	SETBP1	hsa_circ_0108457	-2.33123	1.94E-07
chr10	128768965	129055693	+	DOCK1	hsa_circ_0020399	-2.37176	3.05E-05
chr6	111493897	111498868	+	SLC16A10	hsa_circ_0130335	-2.41667	4.33E-08
chr7	97820925	97823884	+	LMTK2	hsa_circ_0081207	-2.57021	6.30E-11
chrX	110385323	110416309	+	PAK3	hsa_circ_0139566	-2.6349	1.11E-06
chr10	112640990	112650428	+	PDCD4	hsa_circ_0092663	-2.70557	5.68E-06
chr15	57730182	57754090	+	CGNL1	hsa_circ_0035432	-2.74072	6.10E-07
chr11	57258696	57259335	-	SLC43A1	hsa_circ_0005204	-2.74353	4.62E-12
chr4	72205086	72222904	+	SLC4A4	hsa_circ_0008676	-2.79866	1.99E-07
chr9	79952170	79986065	+	VPS13A	hsa_circ_0087255	-2.89185	2.54E-06
chr1	208383624	208391347	-	PLXNA2	hsa_circ_0002472	-2.89307	3.24E-06
chr1	94953097	94956803	+	ABCD3	hsa_circ_0013235	-2.937	1.04E-06
chr15	57730182	57734676	+	CGNL1	hsa_circ_0035431	-3.12651	2.35E-09
chr17	131558	177370	-	RPH3AL	hsa_circ_0041150	-3.21538	1.04E-07
chr6	73005639	73043538	+	RIMS1	hsa_circ_0132246	-3.22669	1.30E-07
chr12	56628607	56629484	+	SLC39A5	hsa_circ_0026920	-3.52249	3.41E-08
chr1	200012901	200090083	+	NR5A2	hsa_circ_0015814	-3.58425	2.62E-13
chr1	200080329	200090083	+	NR5A2	hsa_circ_0015816	-3.81582	5.92E-10
chr12	72955944	72969322	+	TRHDE	hsa_circ_0099195	-4.07037	5.28E-10

chr: chromosome, FC: fold change.

Table S2. Proteins identified by mass spectrometry analysis

Gene name	Number of proteins	Peptides	Unique peptides	Sequence coverage [%]	Molecular weight [kDa]	Intensity
MGME1	3	7	7	23.8	39.42	177510000
TIAL1	14	7	6	19.8	41.462	200440000
ELAVL1	18	6	6	18.7	36.091	157540000
ANXA2	23	5	5	13.6	38.659	54489000
RFC5	5	5	5	17.6	28.786	21899000
RBMS2	15	7	4	23	37.186	112920000
MSI2	10	4	4	16.2	35.196	37068000
FBL	10	5	4	20.2	24.544	27573000
G3BP1	11	3	3	12.4	52.164	7110700
PTBP1	12	3	3	14.5	36.295	23285000
IGHG1	40	3	3	12.1	36.105	18036000
RPS6	6	3	3	13.7	28.708	35559000
GAPDH	10	3	3	12.7	27.87	12870000
RPS2	16	3	3	15.4	21.154	33214000
H2BC15	25	3	3	19.9	18.804	3489500
LRRC41	6	2	2	2.3	67.458	13021000
SFPQ	5	2	2	6.9	55.469	5409000
ARPC1B	5	2	2	8.3	40.949	6010200
RBMS1	11	5	2	14.6	40.147	13787000
TAF15	3	2	2	3.5	40.078	65213000
U2AF1	4	2	2	8.8	27.872	7504300
PHB2	6	2	2	11.3	23.615	5376100
PCBP2	16	2	2	15.2	16.637	1058900
RBM7	8	2	2	16.2	16.436	6252000
TXN	2	2	2	21	11.737	26883000
ZFHX4	2	1	1	0.5	396.21	130570000
CEP350	2	1	1	0.2	350.93	2748300
NEB	4	1	1	0.6	348.96	30527000
INTS1	3	1	1	0.5	266.64	1176700
SPTBN1	5	1	1	0.4	264.44	10837000
MYH10	1	2	1	1.3	229	4502900
MYH9	13	2	1	1.7	226.53	2277000
ECPAS	1	1	1	0.9	223.69	57015000
RESF1	1	1	1	0.7	194.86	11855000
TOPAZ1	1	1	1	0.5	190.92	61418000
NES	2	1	1	0.7	177.42	3738500
MUC2	5	1	1	1.1	153.37	1241500
INSRR	1	1	1	1.5	143.72	6782400
EDRF1	1	1	1	1.1	138.53	48702000

Gene name	Number of proteins	Peptides	Unique peptides	Sequence coverage [%]	Molecular weight [kDa]	Intensity
TTF2	1	1	1	0.9	129.59	7637800
NCKAP1	1	1	1	1	128.79	8084000
GRIN3A	2	1	1	1.4	125.59	28347000
LIFR	2	1	1	0.9	123.83	107550000
TP53BP1	5	1	1	0.7	111.66	1763900
ERN1	1	1	1	1.7	109.73	1250000
INPP4A	2	1	1	1.5	105.09	78785000
NLRP12	1	1	1	1.3	101.73	805940
MYRIP	1	1	1	1.5	95.705	96313000
IQCA1	1	1	1	1	95.34	1247600
GAREM2	1	1	1	0.8	92.881	27054000
UFL1	1	1	1	2	89.594	2897100
C3orf20	2	1	1	1	88.029	7115800
TTLL11	1	1	1	1.1	87.611	37739000
CCDC18	5	1	1	2.2	84.35	819130
TBC1D31	4	1	1	2.4	73.395	760230
ADGB	3	1	1	2.1	70.086	24911000
TRANK1	5	1	1	1.1	69.961	5278800
NLRX1	2	1	1	3.1	67.044	535180
PRSS56	1	1	1	1.3	64.596	12368000
TAP1	13	1	1	2.6	59.939	3282200
PLEKHD1	1	1	1	1.6	59.203	1168100
DDX1	5	1	1	3.6	59.196	1278800
DKF	6	1	1	1.5	57.752	17123000
MFAP1	1	1	1	3.2	51.958	290470000
TRIM29	1	1	1	2.9	50.829	88219000
G3BP2	4	1	1	3.8	50.816	537480
ZNF355P	1	1	1	3.7	49.689	1045000
ILDR2	3	1	1	4.3	48.797	371780
AEBP2	2	1	1	11	46.337	2040900
KLF12	1	1	1	1.7	44.239	5313100
PSD4	4	1	1	2.4	42.188	4727500
ACTBL2	1	2	1	6.6	42.003	8140700
MCPH1	17	1	1	6	41.883	5733600
GDF9	2	1	1	5.2	41.844	1226300
HNRNPK	6	1	1	3.2	41.807	1105900
NIPAL3	2	1	1	2.2	40.873	28262000
OPN1MW3	7	1	1	3.6	40.604	43262000
ULK3	4	1	1	2	40.011	92715000
AKAP8	3	1	1	2.2	39.938	8485000

Gene name	Number of proteins	Peptides	Unique peptides	Sequence coverage [%]	Molecular weight [kDa]	Intensity
PARP11	1	1	1	5.6	39.596	1932100
HPF1	1	1	1	4.3	39.436	4470900
DZIP1	2	1	1	2.9	38.188	1107200
RARB	9	1	1	2.7	37.991	3027600
OR4D10	2	1	1	2.6	35.3	1714000
OR8J2	1	1	1	3.2	35.159	185740000
SZT2	2	1	1	9.8	34.841	10602000
FBLL1	1	2	1	6.6	34.803	6456800
B3GALT6	2	1	1	4.3	34.489	767110
C12orf42	2	1	1	6.5	31.952	268160
PDXP	2	1	1	5.4	31.698	14390000
HVCN1	2	1	1	5.5	31.653	571870
SECISBP2	5	1	1	4.7	30.492	18854000
ADGRB2	8	1	1	4.2	29.196	62087000
SPIC	1	1	1	3.2	29.18	66000000
PEX11B	2	1	1	3.5	28.431	7075600
NELL2	7	1	1	6.5	27.74	904320
SF3B1	2	1	1	6.6	27.221	32534000
EMD	2	1	1	7.8	24.938	9846600
LARP7	2	1	1	4.2	24.488	15771000
MAGT1	4	1	1	4.1	24.378	1700000
RDH13	5	1	1	5.9	24.047	1733200
TFPI	4	1	1	9	23.458	665400
MRPS27	4	1	1	3.6	23.197	5850700
STAU2	11	1	1	8.3	22.799	661910
NAIP	5	1	1	5.1	22.667	8129700
ZC3H18	5	1	1	8.1	21.751	1302700
TUBB2B	30	1	1	9.6	20.598	5271400
APOBEC3A	7	1	1	4	20.168	981860
TAPBPL	4	1	1	5.9	20.041	33012000
ANK2	23	1	1	6.1	19.584	107260000
CCNG1	1	1	1	6.6	19.415	17425000
CPAMD8	1	1	1	6.3	18.876	2711100
MLEC	4	1	1	8.2	16.729	660030
CYB5R3	4	1	1	7.5	16.696	6413300
RPLP0	12	1	1	7.8	16.68	1060900
RAA1	2	1	1	8.2	16.55	125700000
PSMD14	3	1	1	8.4	16.532	590490
VIM	6	1	1	6.7	16.425	6903000
TIA1	5	2	1	16	15.821	5176500

Gene name	Number of proteins	Peptides	Unique peptides	Sequence coverage [%]	Molecular weight [kDa]	Intensity
RABGGTA	6	1	1	5.1	15.673	86554000
C11orf45	2	1	1	13.1	15.559	196370
CENPK	5	1	1	9.8	15.422	13525000
ATP6AP2	17	1	1	6.2	14.069	778810
FRG1	2	1	1	11.2	12.516	1731700
TNNT1	8	1	1	12.5	11.878	13674000
CSF2RB	1	1	1	7.4	11.645	674740000
DCD	1	1	1	10	11.284	7232400
HNRNPH1	13	1	1	17	11.181	1017300
FBXO17	4	1	1	7.4	10.699	11759000
GRK6	1	1	1	8	9.802	586230000
COQ6	1	1	1	20.9	9.3586	30600000
WDR63	1	1	1	19.4	8.4121	208100000
NFATC1	1	1	1	9.9	7.7786	839310
C11orf91	2	1	1	22.9	7.3923	712530000
PABPC4	19	1	1	35.3	7.1041	1897700
HNRNPDL	1	1	1	31.6	6.7215	722380
CCKBR	3	1	1	20	6.288	16990000
WNK1	1	1	1	21.1	6.2173	2984000
HFE	1	1	1	32.7	5.9978	351860
TPM3	10	1	1	22.4	5.8054	975920
PARP8	13	1	1	26.7	5.3553	62020000
SPATA7	14	1	1	16.7	4.7625	14719000

Table S3. Association of circPRKD3 expression with clinicopathological features in PDAC patients

Characteristics	n	circPRKD3 expression		*P* value
		Low	High	
Age (Years)				
≤60	31	18	13	0.808
>60	36	22	14	
Sex				
Male	42	25	17	0.999
Female	25	15	10	
Tumor size (mm)				
≥30	52	27	25	0.018*
<30	15	13	2	
TNM stage				
I-II	60	34	26	0.227
III-IV	7	6	1	
Degrees of differentiation				
Low	20	10	10	0.415
Moderate	47	30	17	
Lymph node metastasis				
Positive	22	7	15	0.002*
Negative	45	33	12	

Table S4. Serum levels of circPRKD3 and conventional tumor markers in PDAC patients and controls

Sample Name	Disease	Gender	Age	CircPRKD3	CEA	CA19-9	CA125
P671	PDAC	Female	55	0.932911	2.24	1000	182
P675	PDAC	Female	50	3.096293	7.52	245	13.9
P680	PDAC	Male	32	3.081519	3.48	595	9.58
P712	PDAC	Male	51	0.980385	32.2	1000	71.6
P726	PDAC	Male	66	2.979642	1.48	215	15
P731	PDAC	Male	64	5.128865	20.8	31.8	525
T102	PDAC	Male	53	3.554264	1.9	827	19.8
T104	PDAC	Female	58	1.866142	3.44	54.3	9.08
T11	PDAC	Female	63	0.940965	3.78	276	19.4
T111	PDAC	Female	75	4.154561	1.87	251	13.5
T114	PDAC	Male	49	6.784458	4.89	1000	16.2
T115	PDAC	Female	69	2.981494	1.78	268	27.1
T116	PDAC	Male	84	2.382505	3.52	763	424
T118	PDAC	Female	64	2.224739	7.7	1000	9.42
T119	PDAC	Male	42	1.887684	0.81	42.9	7.65
T123	PDAC	Female	58	1.42518	5.91	536	171
T129	PDAC	Male	65	5.052579	2.73	2	7.34
T132	PDAC	Male	49	2.416359	6.75	166	13.2
T14	PDAC	Male	31	2.243761	5.48	807	21.5
T142	PDAC	Female	57	2.497498	11.2	447	44.4
T145	PDAC	Male	58	2.192195	4.21	336	18.7
T15	PDAC	Male	68	1.526649	2.5	6.66	12.7
T182	PDAC	Male	60	0.092782	2.67	88.5	11.3
T20	PDAC	Male	51	0.23003	1.72	10.6	7.71
T25	PDAC	Female	77	1.013002	2.22	734	21.6
T34	PDAC	Male	50	1.749336	32.3	1000	71.6
T38	PDAC	Male	70	0.659327	3.94	244	27.5
T39	PDAC	Male	49	0.922911	1.04	15.2	48.1
T45	PDAC	Male	57	3.31599	3.31	15.7	12.2
T47	PDAC	Male	49	1.023608	22.3	44.9	407
T49	PDAC	Male	47	1.420034	2.66	849	73.8
T50	PDAC	Male	62	2.801568	3.44	26.7	8.59
T59	PDAC	Male	71	2.605246	2.24	42	8.58
T73	PDAC	Male	67	1.041743	5.51	203	9.45
T77	PDAC	Female	62	2.67962	15.3	710	32.1
T78	PDAC	Male	67	3.062823	49.2	32.2	46
T97	PDAC	Male	62	1.793827	2.78	411	25.6
T98	PDAC	Male	54	0.632685	2.32	66.3	6.98
P677	Pancreatic pseudocyst	Female	44	1.549485	2.4	19.8	14.6

P681	Pancreatic pseudocyst	Male	54	0.471168	2.7	6.53	75.5
T36	Pancreatic pseudocyst	Male	50	0.195205	1.07	6.85	62.2
T67	Pancreatic pseudocyst	Male	41	1.697578	4.07	6.8	125
T76	Pancreatic pseudocyst	Female	54	1.652043	1.78	7.73	14.7
P676	Chronic pancreatitis	Male	71	1.206723	8.78	10.7	10.4
T109	Chronic pancreatitis	Female	69	5.203205	2.31	84.7	9.91
T27	Chronic pancreatitis	Male	51	0.828809	2.51	11	8.86
T30	Chronic pancreatitis	Male	45	0.428857	1.49	12.3	51.8
T31	Chronic pancreatitis	Male	64	0.257316	1.78	20.7	13.4
T37	Chronic pancreatitis	Male	68	1.305469	1.46	40.6	6.66
T51	Chronic pancreatitis	Male	56	0.204959	1.8	37.4	12.5
T79	Chronic pancreatitis	Male	78	4.340038	1.33	5.41	31.9

Table S5. Primer and shRNA sequences used for plasmid construction

Name	Primers or shRNA sequences (5'-3')
WT circPRKD3	ATTTTTTTATTTTATGCAGGCTAACTATATGTCAGAAAGCATCAGC (Forward)<br>TTGGAATTTTGAATACTTACCTTTTGATAAACTATGGAGCACACAA (Reverse)
circPRKD3 GT to CC linear transcript mutant	GCTCCATAGTTTATCAAAAGCCAAGTATTCAAAATTCCA (Forward)<br>TGGAATTTTGAATACTTGGCTTTTGATAAACTATGGAGC (Reverse)
circPRKD3 AG to TT linear transcript mutant	TTTTTATTTTATGCTTGCTAACTATATGTCAGAAAGC (Forward)<br>GCTTTCTGACATATAGTTAGCAAGCATAAAATAAAAAA (Reverse)
circPRK3 t459a_g461c MSI2-binding site mutant	CATATAGTTAGCCTTTTGATAAAGTTTGGAGCACACAAGATCCTTGACA (Forward)<br>TGTCAAGGATCTTGTGTGCTCCAAACTTTATCAAAAGGCTAACTATATG (Reverse)
CircPRK3 t657a_g659c MSI2-binding site mutant	TCAGTACTTTTCCTTTGGTTTAGTTATTTGATAGGACACCTTCTCAAATGTC (Forward)<br>GACATTTGAGAAGGTGTCCTATCAAATAACTAAACCAAAGGAAAAGTACTGA (Reverse)
Flag-MSI2 full length vector (FL)	GCTATCTAGAGCCACCATGGAGGCAAATGGGAGCCAAGG (Forward)<br>GCTAGGATCCTCACTTATCGTCGTCATCCTTGTAATCATGGTATCCATTTGTAAAGGC (Reverse)
Flag-MSI2 T1 truncate mutant	GCTAGGATCCTCACTTATCGTCGTCATCCTTGTAATCCGGCTGAGCTTTCTTACATTC (Reverse)
Flag-MSI2 T2 truncate mutant	GCTAGGATCCTCACTTATCGTCGTCATCCTTGTAATCTTTCTTTGTTCTTGTGACCATCTT (Reverse)
Flag-MSI2 T3 truncate mutant	GCTATCTAGAGCCACCATGAAGAAAATATTTGTAGGCGGGTTATCTGCG (Forward)
Flag-MSI2 T4 truncate mutant	GCTATCTAGAGCCACCATGGGTAAAATGTTTATCGGTGGACTGAGC (Forward)
Flag-MSI2 K22A mutant	AGCACGACCCCGGTGCAATGTTTATCGGTGGACTGAGC (Forward)<br>CCACCGATAAACATTGCACCGGGGTCGTGCTGGGAGTC (Reverse)
Flag-MSI2 R100A mutant	GTTGCATTTCCTCGTGCAGCGCAACCCAAGATGGTCAC (Forward)<br>TCTTGGGTTGCGCTGCACGAGGAAATGCAACTTTGGG (Reverse)
CircPRKD3 shRNA (Sh-circ)	AAAAGGCTAACTATATGTCAG
sh-MSI2#1 shRNA	GTGGAAGATGTAAAGCAATAT
sh-MSI2#2 shRNA	CCCAACTTCGTGGCGACCTAT
sh-BTRC#1 shRNA	GCGTTGTATTCGATTTGATAA
sh-BTRC#2 shRNA	GCTGAACTTGTGTGCAAGGAA
sh-DBC2#1 shRNA	GCCAAACGTAGAGACCATCAA
sh-DBC2#2 shRNA	GCACCAACTACAACAACGTGT
sh-HUWE1#1 shRNA	CGACGAGAACTAGCACAGAAT
sh-HUWE1#2 shRNA	GCTCCCACTATAACCTCACTT
Scr shRNA	TTCTCCGAACGTGTCACGTTT

Table S6. Primer sequences used for RT-PCR and RT-qPCR analysis

	Targets	Forward primer (5'-3')	Reverse primer (5'-3')
RT-PCR	Divergent primers	ACCATCACTCACCAACTCCA	AATGTCCACACCTTAAATCACC
	Convergent primers	GAGAAATAGGACCTTTGGGGC	TCTTAGGACTTGAACACGGAGAA
	PRKD3 mRNA	CAAGACCCCTACAGCCTGAATA	GTCATCCAAACCCCGACTACT
	Linear transcript	TAGAGTAGGCGAGGACAG	TCAGCAGGATAGAGTTGAC
RT-qPCR	circPRKD3	GTGTTACCATTGAAGCCCAGG	TGGGTCCATCGAGAAAAGCT
	PRKD3 mRNA	CAGATGAGGTGCTTGGTTCAG	GCCACTTCATTACGGAGTTGAC
	Actin mRNA	AGGCCAACCGCGAGAAGATG	GCCAGAGGCGTACAGGGATA
	U6 snRNA	CGCTTCGGCAGCACATATAC	AGGGGCCATGCTAATCTTCT
	MSI2 mRNA	GTTCGCAGACCCAGCAAGTGT	TGTTCGCAGATAACCCGCCTAC
	MYC mRNA	CTTCTCTGAAAGGCTCTCCTTG	GTCGAGGTCATAGTTCCTGTTG
	TUBA1B mRNA	GCCCTACAACTCCATCCTCA	GTCAACATTCAGGGCTCCAT
	FLNA mRNA	GCGTCCAAGGTCAAGTGCTCTG	GTGCCATCAGCGTTGTCTACCA
	ITGB1 mRNA	AACAGAACTGCACCAGCCCATT	ACCAGCAGCCGTGTAACATTCC
	LAMB3 mRNA	GCCGAGCCTGTGACTGTGATT	GGAGGTCCGCATCATAGGTCTG
	PXN mRNA	ATTCATCCACCAGCAGCCTCAG	GCACGGAGAGCCAACACTGT

Table S7. Oligonucleotide sequences for RNA pull-down and FISH assays

The primers for *in vitro* cyclization (5'-3')	
Sense splint	GATGCTTTCTGACATATAGTTAGCCTTTTGATAAACTATGGAGCAC
Antisense splint	GTGCTCCATAGTTTATCAAAAGGCTAACTATATGTCAGAAAGCATC
Sense-F	TAATACGACTCACTATAGGCTAACTATATGTCAGAAAGCA
Sense-R	TTTTGATAAACTATGGAGCACAC
Antisense-F	TAATACGACTCACTATAGCCTTTTGATAAACTATGGAGC
Antisense-R	TAACTATATGTCAGAAAGCATCAGC
Biotin or CY3 labelled probes in pull down and FISH assays (5'-3')	
Sense DNA probe (3' biotin)	CTGACATATAGTTAGCCTTTTGATAAACTATGG
Antisense DNA probe (3' biotin)	CCATAGTTTATCAAAAGGCTAACTATATGTCAG
CircPRKD3 FISH probe (5' CY3)	CTGACATATAGTTAGCCTTTTGATAAACTATGGAGC
U6 FISH probe (5' CY3)	GCTAATCTTCTCTGTATCGTTCCAATTTTAGTATATGTGCTGCCG
18s FISH probe (5' CY3)	CATGGCTTAATCTTTGAGAC
P1 RNA probe (5' biotin)	GUGUGCUCCAUAGUUUAUCAAAAG
P2 RNA probe (5' biotin)	UCCUAUCAAAUUAGUAAACCAAAG
P1-mut RNA probe (5' biotin)	GUGUGCUCCAAACUUUAUCAAAAG
P2-mut RNA probe (5' biotin)	UCCUAUCAAAUAACUAAACCAAAG
SCR probe (5' biotin)	UUCUCCGAACGUGUCACGUUU

Uncropped Immunoblot Images

Figure 1G

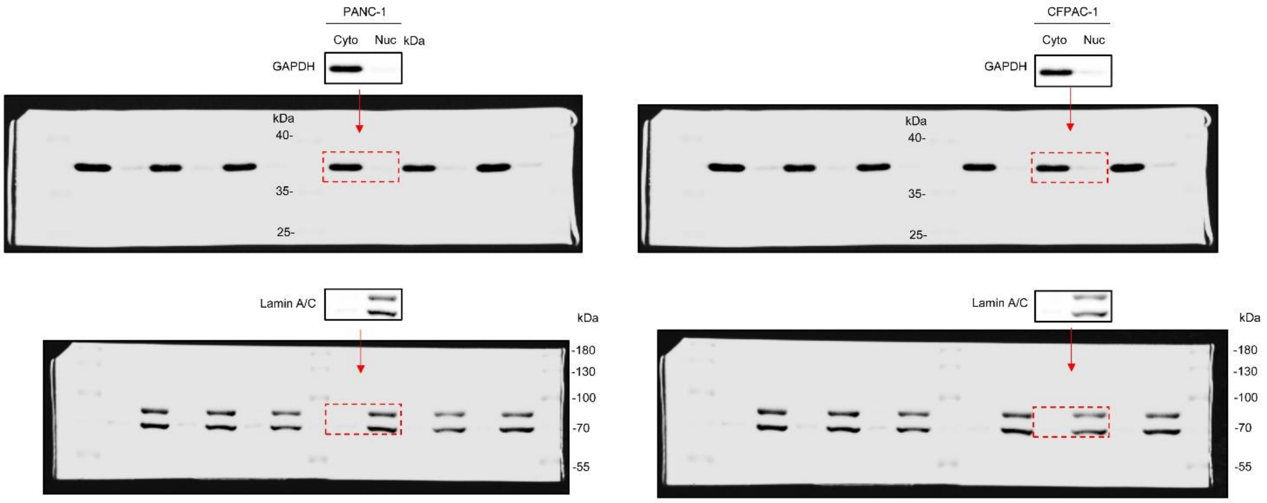

Figure 4C

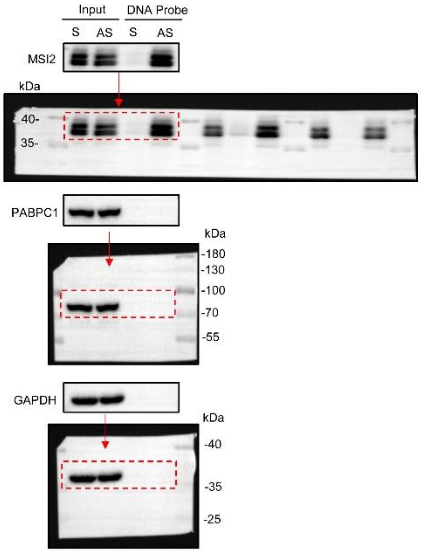

Figure 4D

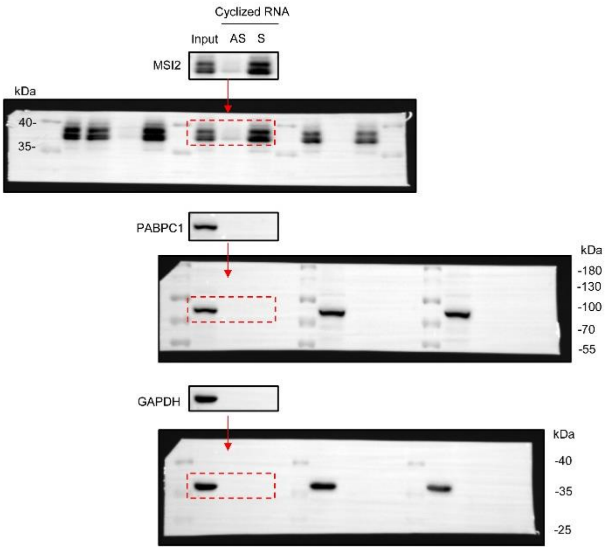

Figure 4E

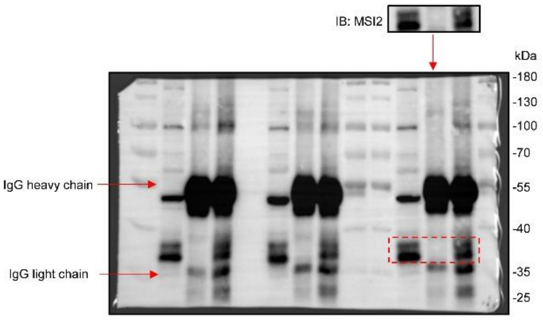

Figure 4G

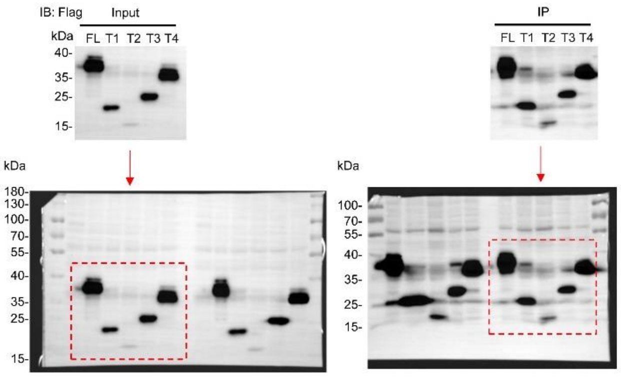

Figure 4I

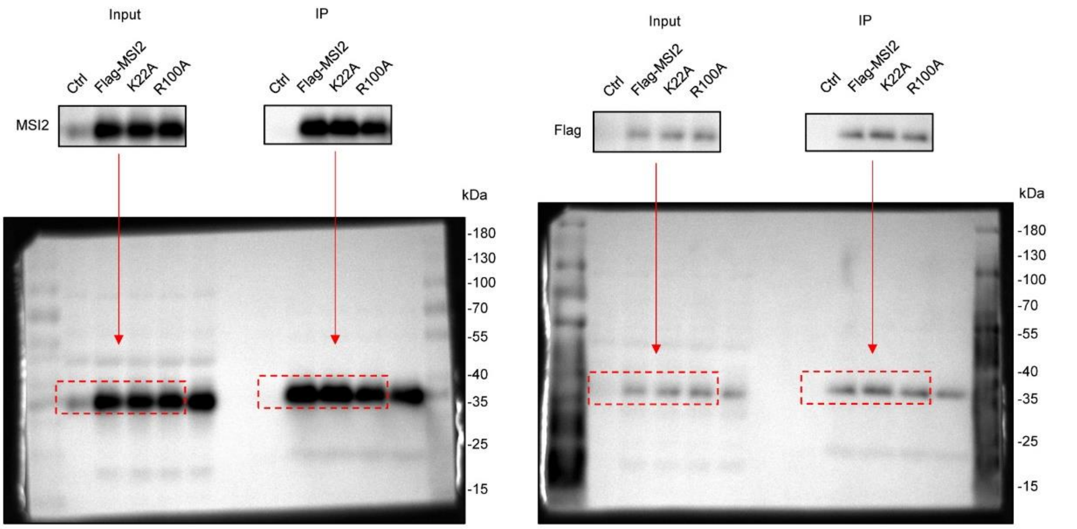

Figure 5B

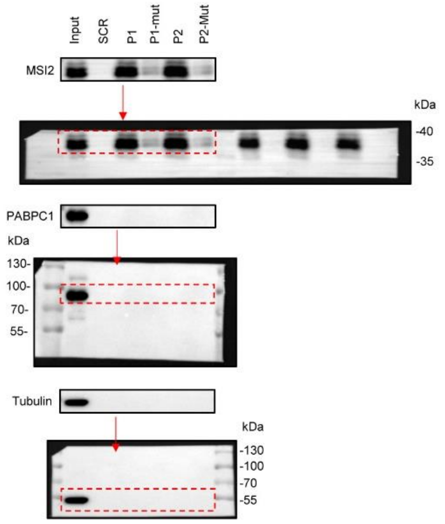

Figure 5C

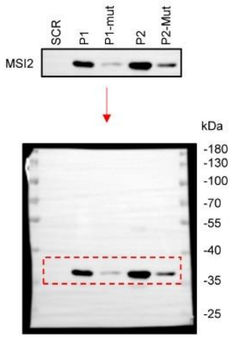

Figure 5E

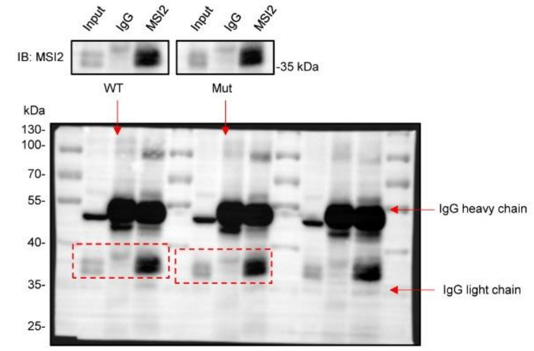

Figure 6A

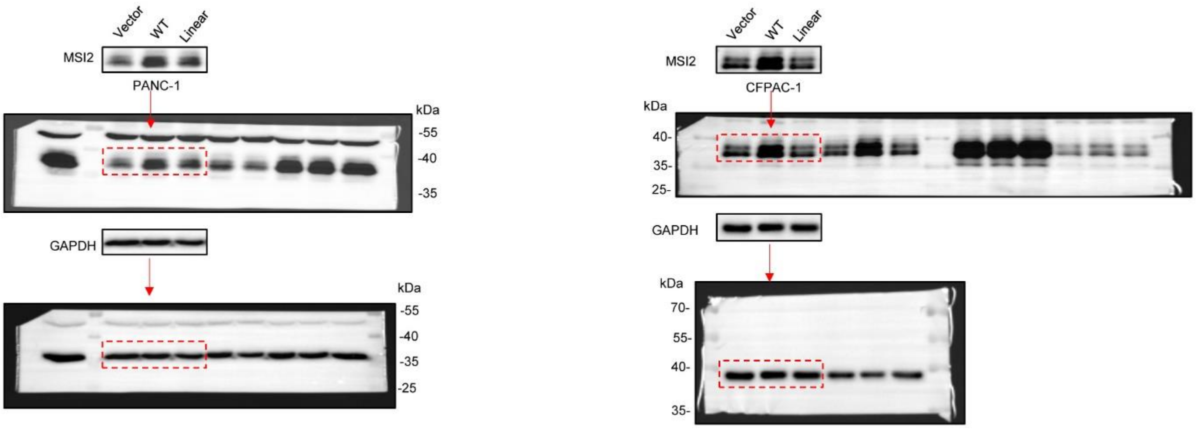

Figure 6B

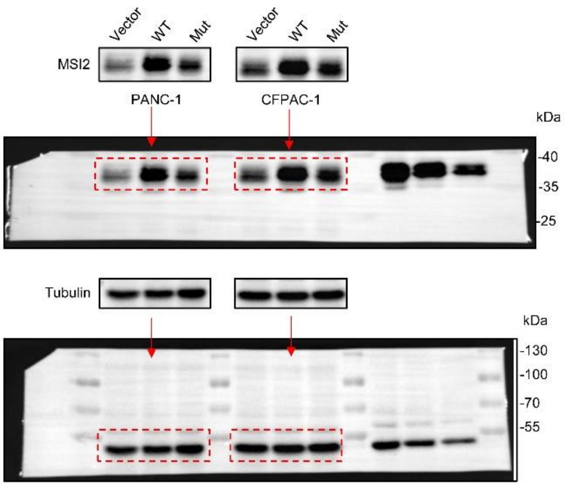

Figure 6C

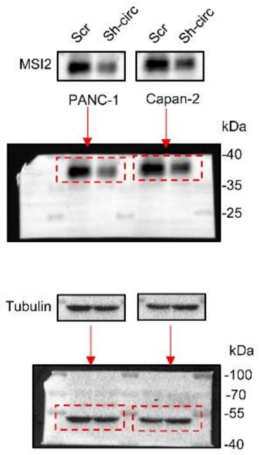

Figure 6F

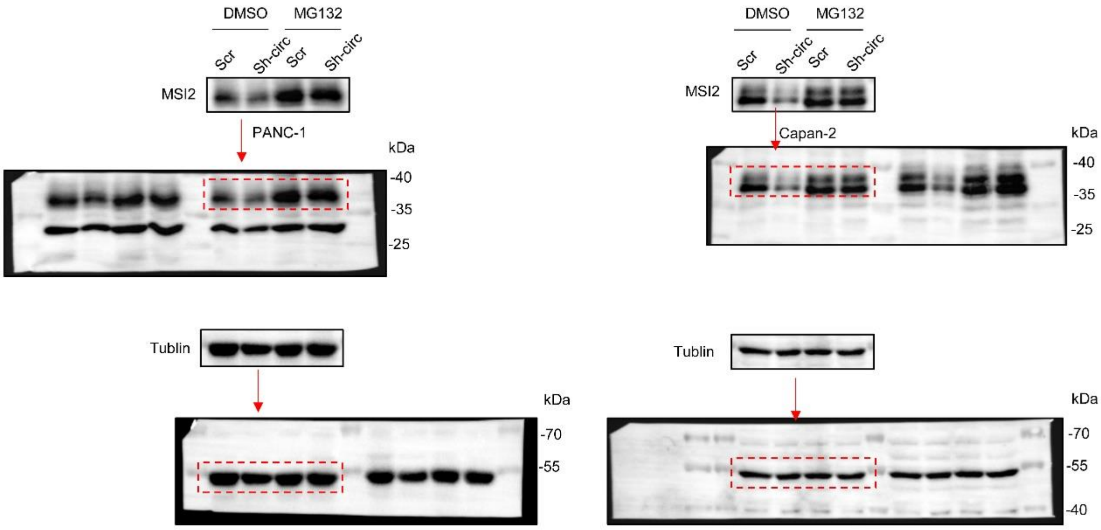

Figure 6G

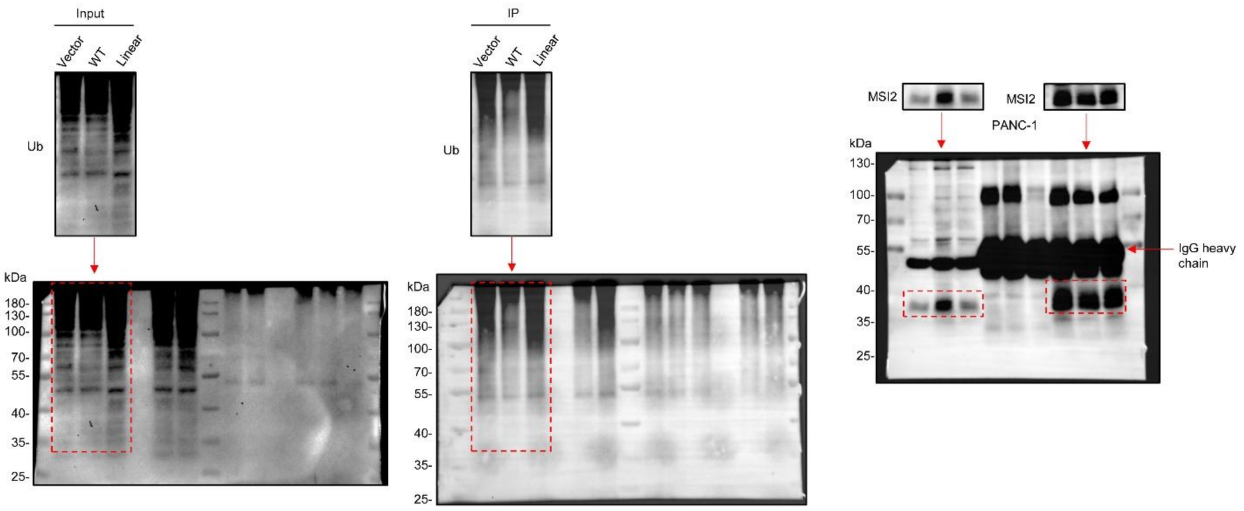

Figure 6H

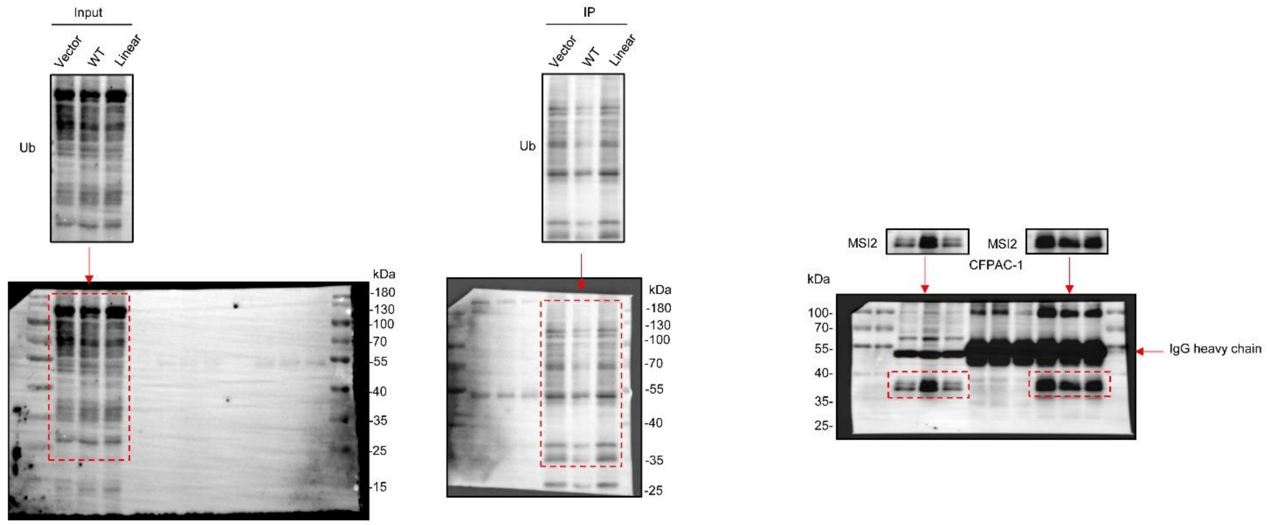

Figure 6I

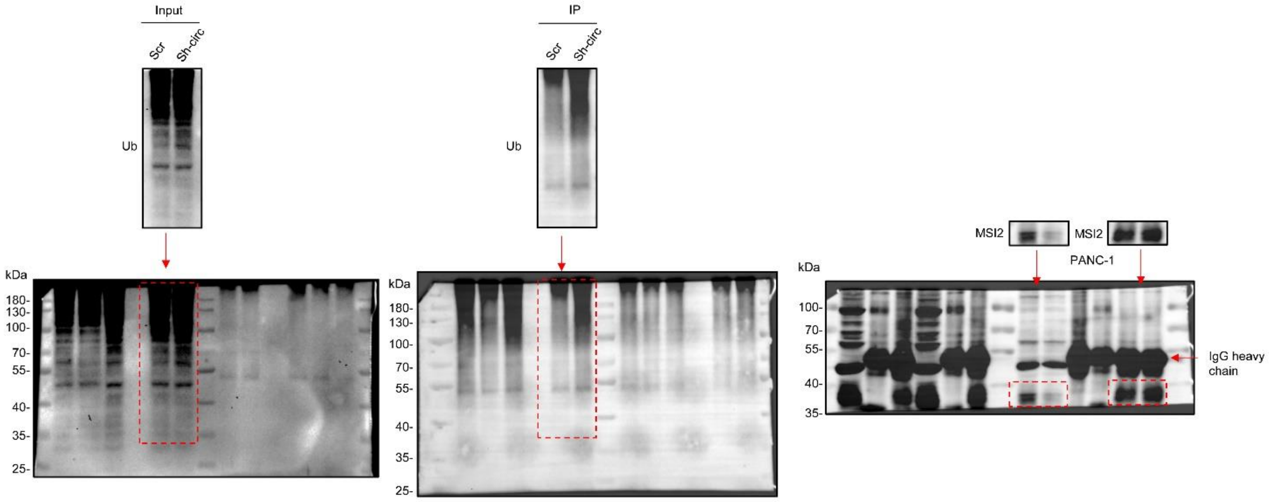

Figure 7A

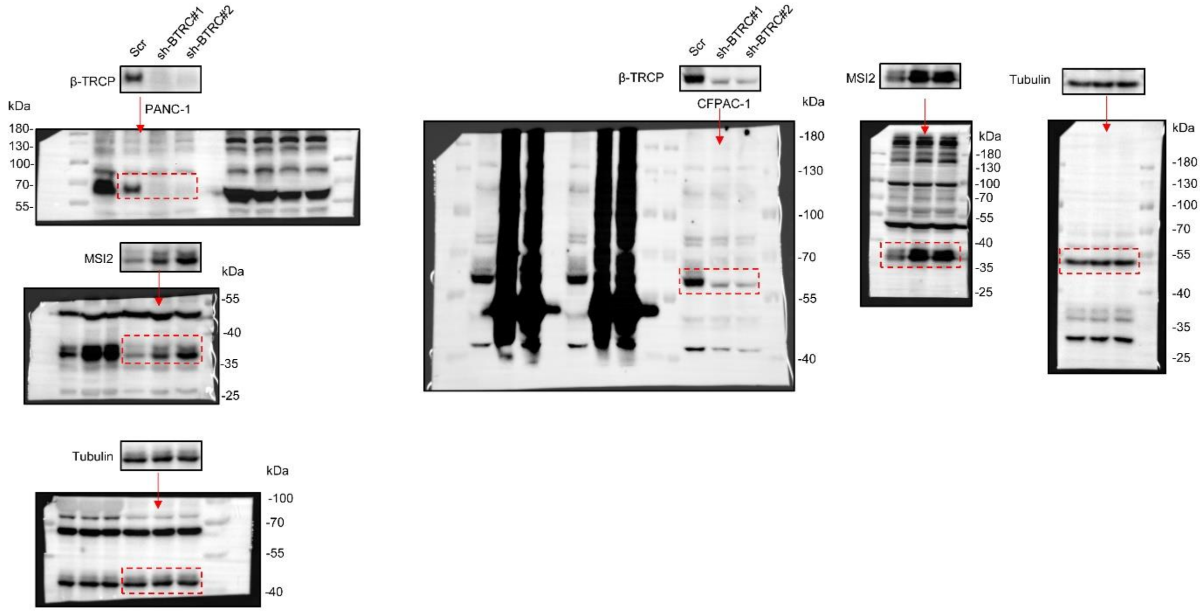

Figure 7B

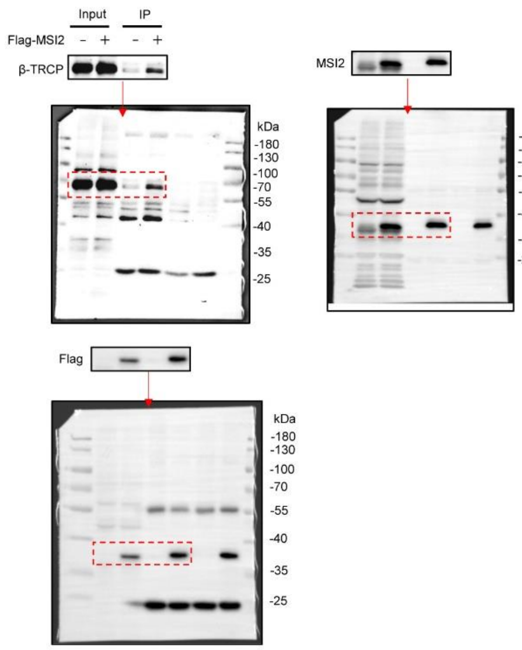

Figure 7C

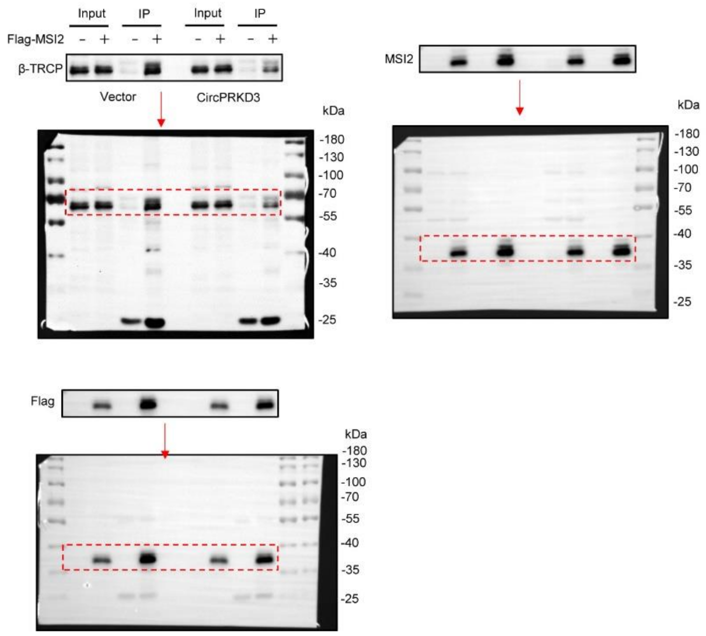

Figure 7D

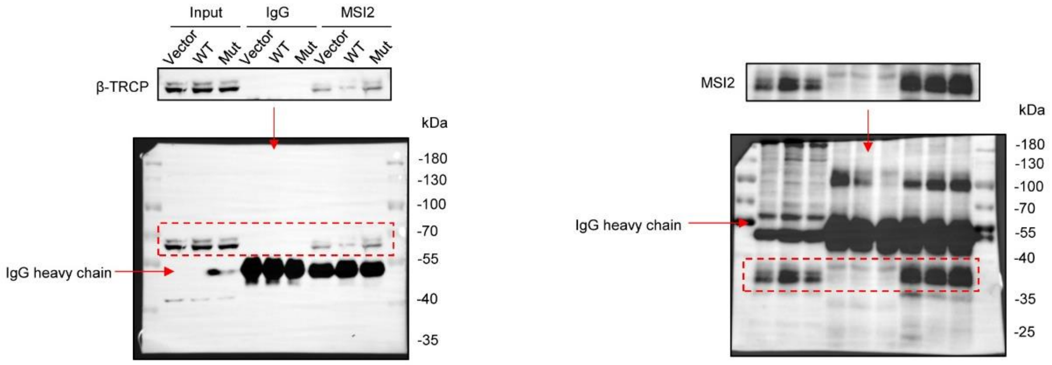

Figure 7E

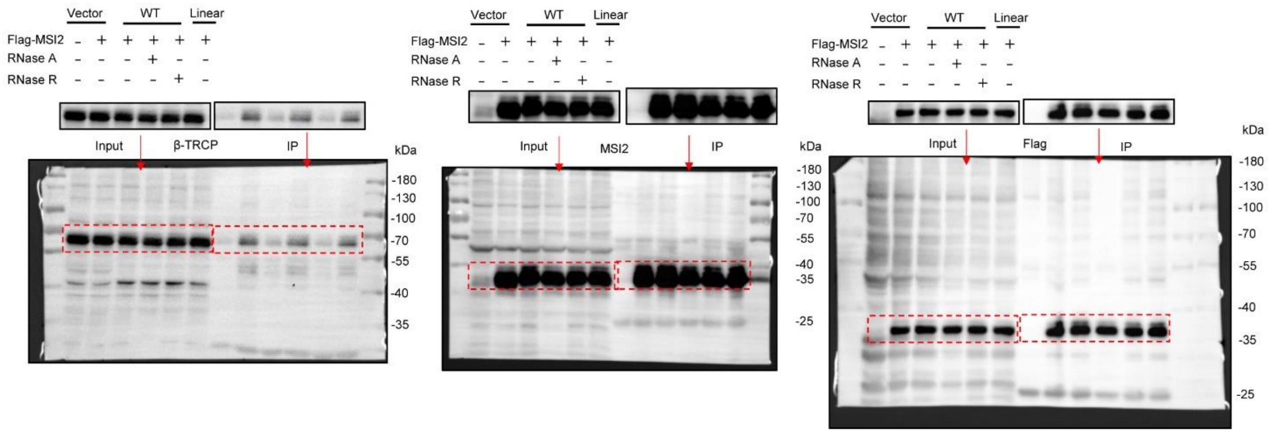

Figure 7F

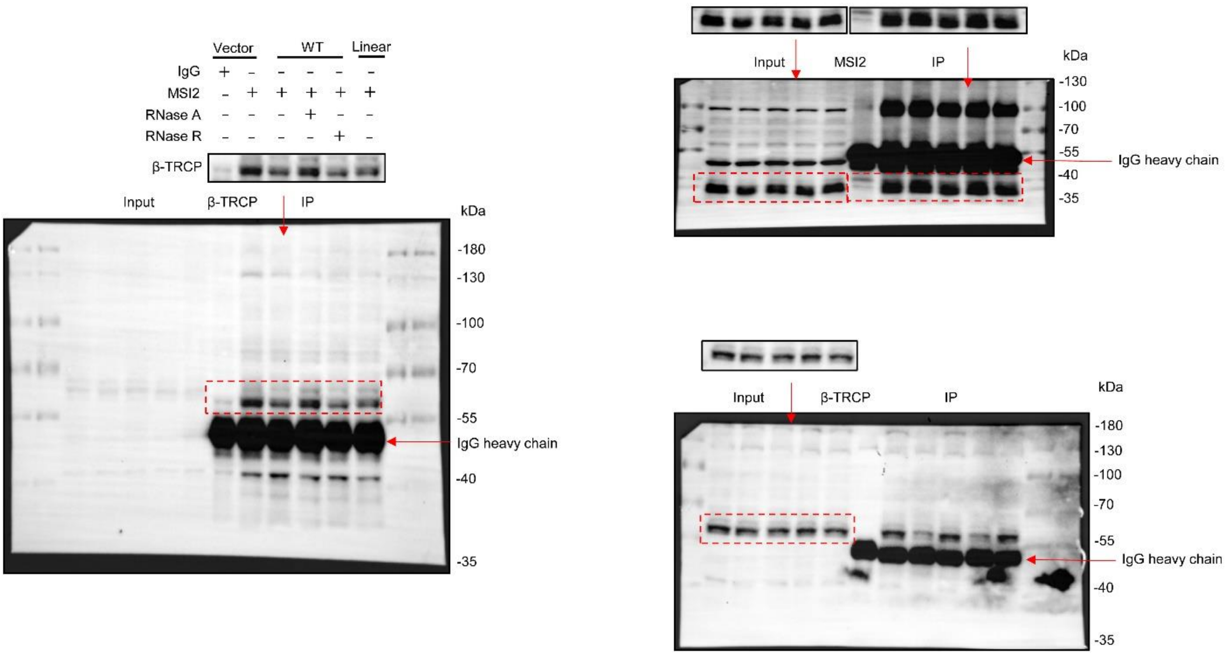

Figure 7G

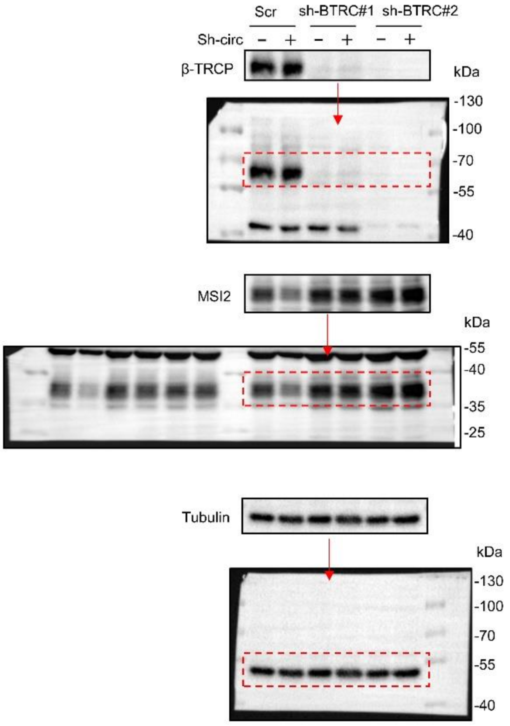

Figure 8A

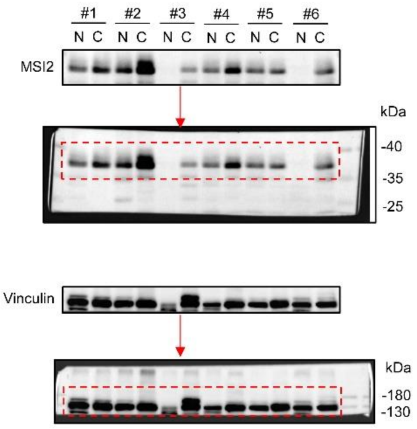

Figure S1D

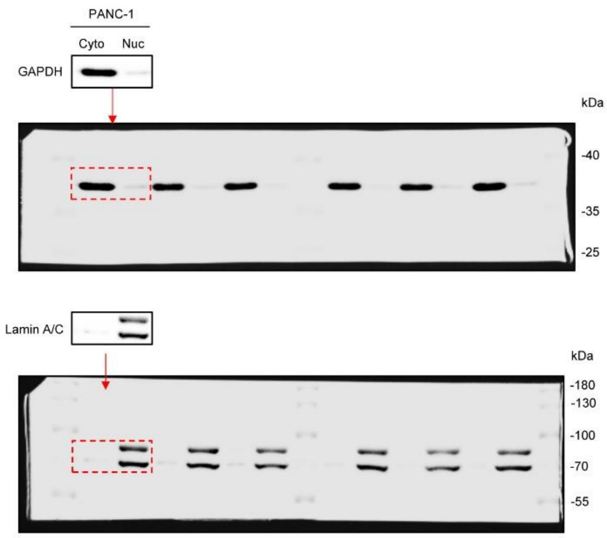

Figure S1E

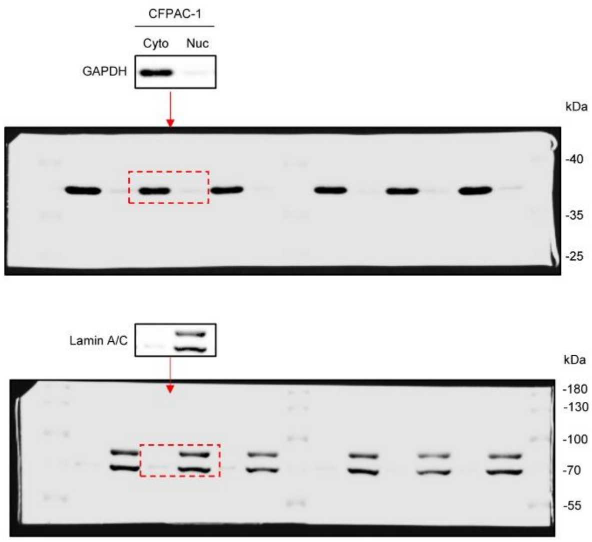

Figure S2E

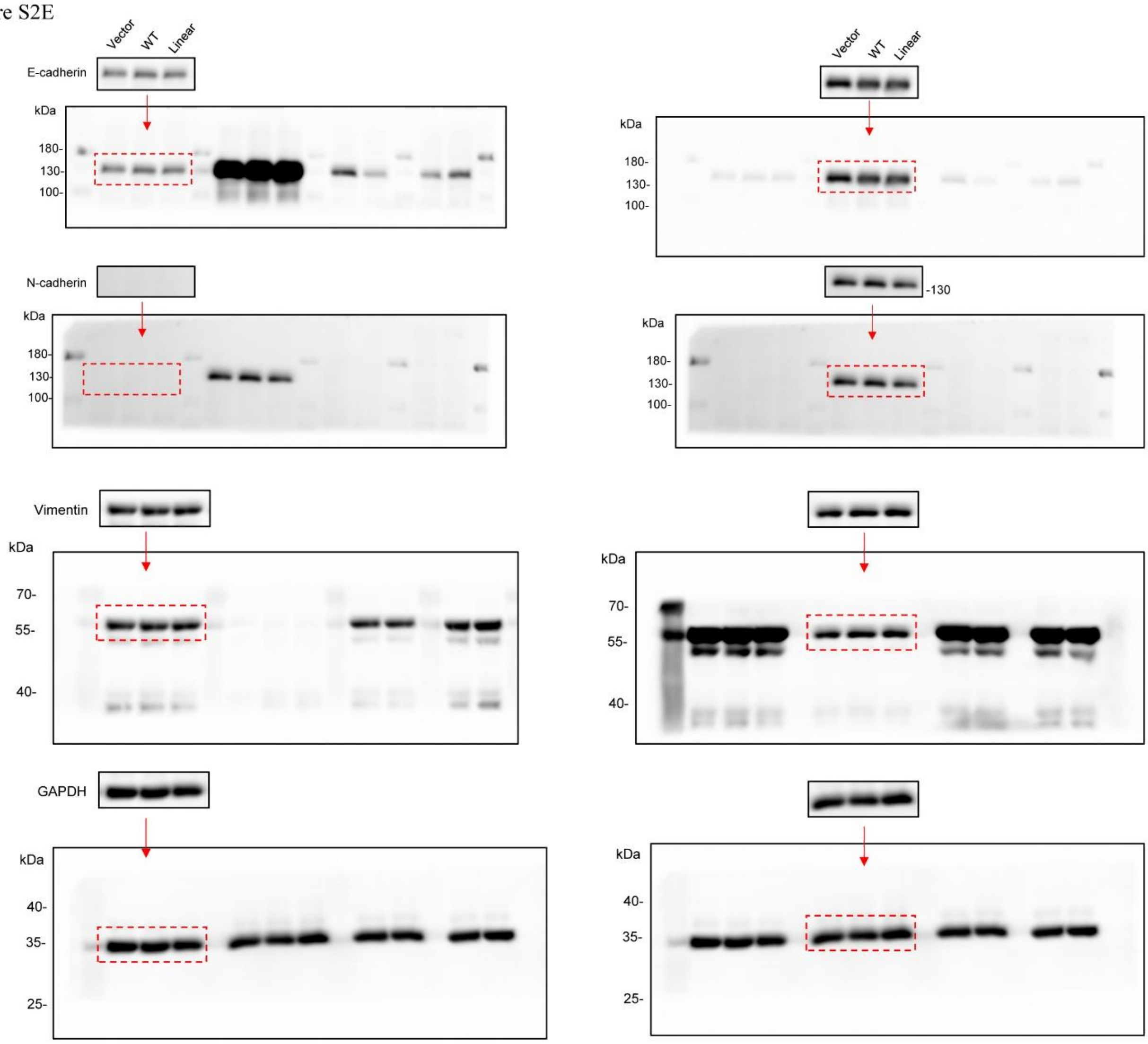

Figure S3D

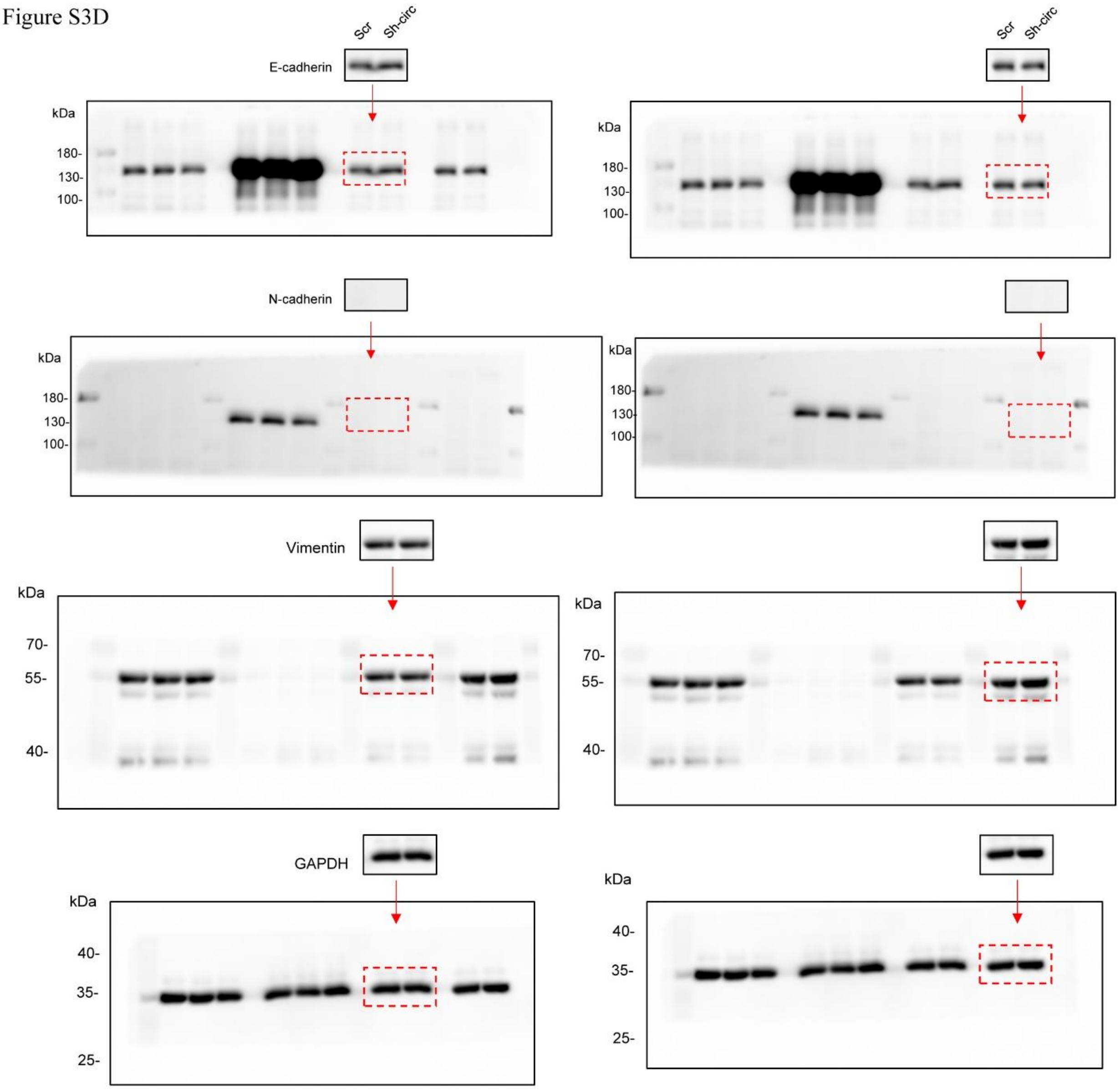

Figure S4C

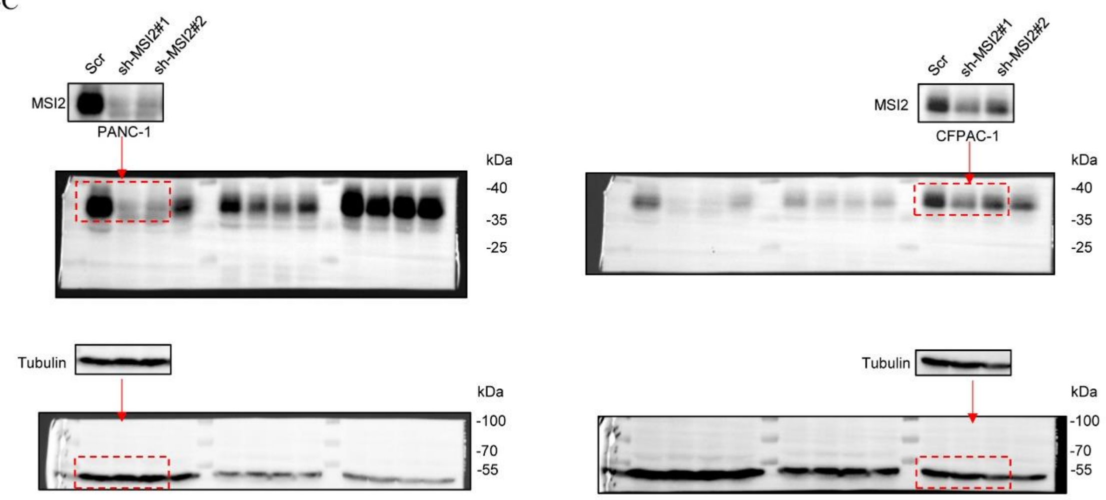

Figure S6A

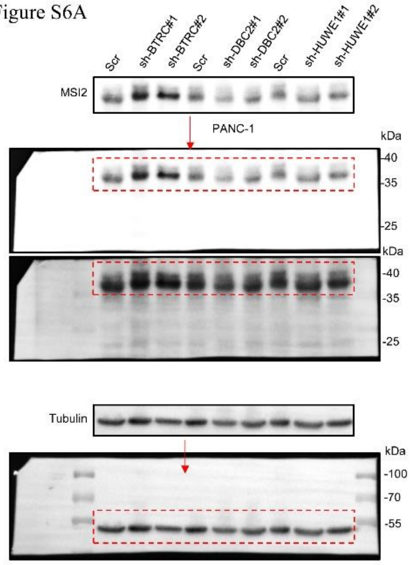

Figure S6B

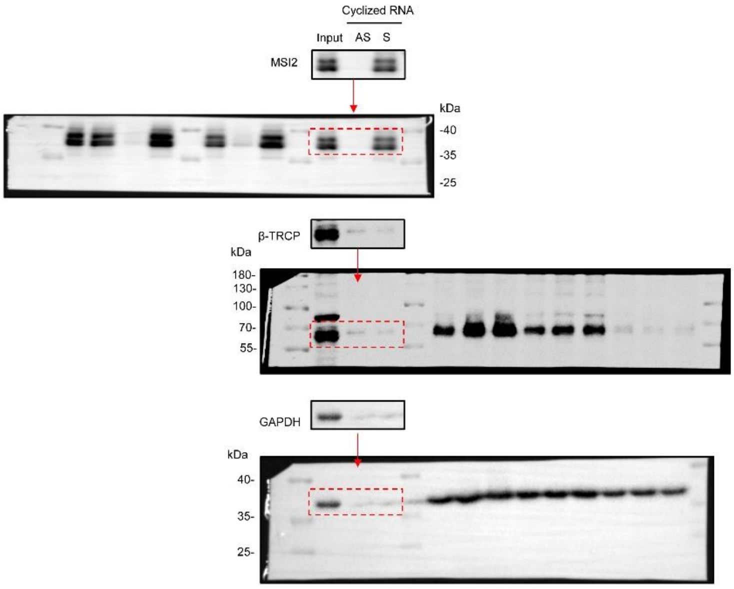

Figure S7A

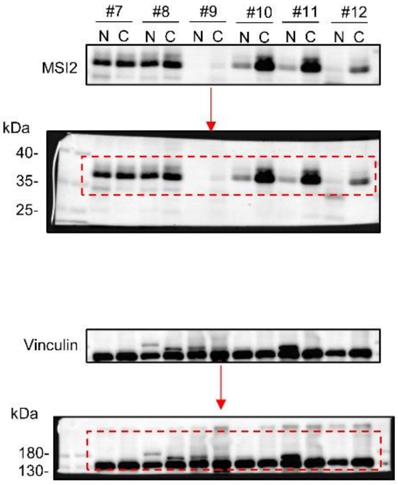

Figure S7B

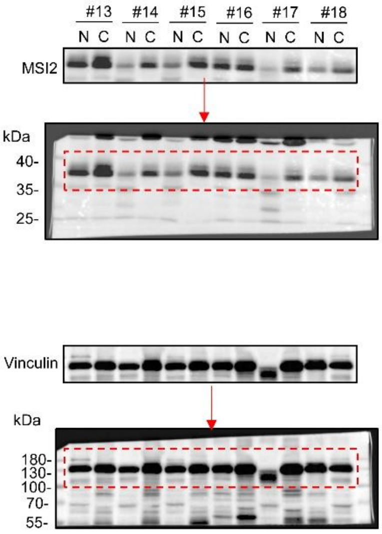

Supplement: Supplementary 1 — Figs. S1 to S7 Tables S1 to S7 Uncropped immunoblot images [file research.0918.f1.pdf]
